# Group‐level versus individualized approaches for capturing tau PET changes and clinical progression in Alzheimer's disease

**DOI:** 10.1002/alz.71718

**Published:** 2026-08-19

**Authors:** Hannah de Bruin, Colin Groot, Zeyu Zhu, Elsmarieke van de Giessen, Sergey Shcherbinin, Vikas Kotari, Diana O. Svaldi, Yolande A. L. Pijnenburg, Emma M. Coomans, Rik Ossenkoppele, Nicolai Franzmeier, for the Alzheimer's Disease Neuroimaging Initiative (ADNI)

**Affiliations:** ^1^ Alzheimer Center Amsterdam, Neurology Vrije Universiteit Amsterdam, Amsterdam UMC location VUmc Amsterdam the Netherlands; ^2^ Amsterdam Neuroscience Neurodegeneration Amsterdam the Netherlands; ^3^ Institute for Stroke and Dementia Research (ISD), University Hospital Ludwig Maximilian University of Munich Munich Germany; ^4^ Radiology & Nuclear Medicine Vrije Universiteit Amsterdam, Amsterdam UMC location VUmc Amsterdam the Netherlands; ^5^ Amsterdam Neuroscience Brain Imaging Amsterdam the Netherlands; ^6^ Eli Lilly and Company Indianapolis Indiana USA; ^7^ Munich Cluster for Systems Neurology (SyNergy) Munich Germany; ^8^ The Sahlgrenska Academy, Institute of Neuroscience and Physiology, Psychiatry and Neurochemistry University of Gothenburg Gothenburg Sweden

**Keywords:** Alzheimer's disease, clinical progression, cognitive decline, individualized regions of interest, longitudinal change, tau positron emission tomography

## Abstract

**INTRODUCTION:**

Quantification approaches based on group‐level regions of interest (ROIs) are widely used for evaluating tau positron emission tomography (PET) and clinical progression in Alzheimer's disease (AD), but we hypothesize that personalized approaches better capture inter‐individual heterogeneity.

**METHODS:**

We included 556 amyloid beta–positive individuals from seven cohorts (335 cognitively unimpaired [CU]; 221 cognitively impaired [CI]) with ≥ 2 [^18^F]flortaucipir (tau) PET scans. We compared three group‐level and seven individualized tau PET quantification approaches (and a voxel‐wise spatial extent metric) by (1) percentage change, (2) sensitivity to change, and (3) clinical associations.

**RESULTS:**

Percentage change was highest in a composite temporal ROI (temporal‐meta) in CU and CI. Individualized Braak stage–based (CU) and connectivity‐informed (CU/CI) approaches, and tau spatial extent (CI), performed well for sensitivity to change and clinical associations, but temporal‐meta was consistently a top performer.

**DISCUSSION:**

Contrary to our hypothesis, temporal‐meta quantification was most robustly associated with biological and clinical progression across disease stages, making it well suited for monitoring AD progression with tau PET.

## Highlights

- We used group‐level and individualized tau positron emission tomography regions of interest to track Alzheimer's disease (AD) progression.
- Quantification in the temporal‐meta ROI was most robust across disease stages and use cases.
- Current Braak stage quantification was also informative in preclinical AD.
- The voxel‐wise tau spatial extent (TAU‐SPEX) metric performed well in early symptomatic AD.
- Connectivity‐based Q1 measures were informative in both disease stages.

## 1 BACKGROUND

Alzheimer's disease (AD) is characterized by the accumulation of amyloid beta (Aβ) plaques and hyperphosphorylated neurofibrillary tau tangles.^1^ Tau burden and its progression are more closely linked to neurodegeneration and cognitive decline than Aβ, making tau an important target for monitoring disease progression and evaluating treatment effects.^2^, ^3^, ^4^ Tau positron emission tomography (PET) allows in vivo quantification of regional tau burden, and is increasingly used as an outcome measure in observational studies and clinical trials of disease‐modifying therapies.^5^, ^6^, ^7^

A key challenge is how to best capture tau PET progression across the brain.^8^, ^9^ Most studies use predefined, group‐level “one‐size‐fits‐all” regions of interest (ROIs).^10^, ^11^, ^12^, ^13^ The most widely used framework is Braak staging, which translates *post mortem* staging systems for tau pathology to tau PET imaging and assumes stereotypical progression from medial temporal regions to the neocortex, although in vivo tau‐PET Braak ROIs should not be interpreted as directly equivalent to neuropathological Braak stages as PET likely lags behind.^1^, ^14^, ^15^ Another common approach is to use a composite temporal ROI (e.g., temporal‐meta)^11^, ^16^ that combines multiple medial and lateral temporal lobe regions associated with early‐ to intermediate‐stage tau accumulation in AD. Although practical and interpretable, these approaches assume homogeneous spatial tau progression patterns across individuals and emphasize temporal lobe involvement. However, tau PET distribution and longitudinal change vary markedly between individuals, and differential patterns contribute to clinical heterogeneity.^8^, ^10^, ^17^, ^18^, ^19^, ^20^, ^21^, ^22^ For example, atypical AD phenotypes and early‐onset AD patients often show early and predominant neocortical involvement, potentially rendering temporal lobe–focused ROIs suboptimal for capturing tau change in these populations.^17^, ^19^, ^21^, ^22^ Notably, there is also substantial inter‐individual variability in spatial patterns of tau deposition within typical amnestic AD.^8^ Additionally, commonly used global tau PET measures average signal across large areas of the cortex to provide an overall estimate of tau load.^12^ While this does provide a methodologically convenient solution to tau PET quantification, this subject‐agnostic approach could dilute regional effects when regions with substantial progression are averaged with regions showing little or no change, possibly reducing sensitivity to detect clinically meaningful longitudinal tau change.

Several other, often individualized, ROIs have been proposed to better capture subject‐specific tau patterns and progression.^8^, ^23^, ^24^, ^25^ Prior work suggests that tau accumulation starts in “epicenters” and that tau subsequently progresses to regions functionally connected to these epicenters, consistent with network‐based propagation of tau pathology.^8^, ^23^, ^26^, ^27^, ^28^, ^29^, ^30^, ^31^, ^32^ Accordingly, longitudinal tau PET change can be quantified within epicenters as well as within connectivity‐informed target regions that are most strongly connected to the epicenter.^8^, ^23^, ^25^ Moreover, instead of using fixed, group‐level Braak ROIs, an individualized Braak‐based approach can assign each participant to the predefined Braak ROI corresponding to the highest Braak stage showing tau positivity (i.e., their current Braak stage) and subsequently track tau accumulation in this ROI.^25^ In addition, tau pathology can also be characterized in terms of spatial extent, reflecting how widely tau has spread across the cortex, rather than magnitude within a specific region. Metrics such as fill states have been proposed, which quantify the proportion of abnormal voxels relative to controls or exceeding a certain abnormality threshold, offering a sensitive marker of contemporaneous tau distribution and subsequent tau progression.^33^, ^34^

The overarching aim of this study was to compare group‐level and individualized tau PET target region selections as well as tau spatial extent in their ability to capture biological and clinical AD progression across the syndromic spectrum, that is, from the asymptomatic preclinical stage to early clinical stages. Specifically, we compared group‐level and individualized tau PET quantification approaches in their ability to detect AD progression across three dimensions: (1) longitudinal tau PET percentage change, (2) sensitivity to detect longitudinal tau PET change, and (3) associations with cognitive decline and clinical progression. For (3), we evaluated both baseline tau PET burden and longitudinal tau PET change as predictors of disease progression. Based on the remarkable heterogeneity in tau distribution, and strong clinical correlates of regional tau, we hypothesized that individualized ROI approaches and tau spatial extent would be more effective in capturing AD progression than generic group‐level ROI approaches.

**RESEARCH IN CONTEXT**

1. **Systematic review**: We reviewed existing publications in standard databases to identify cohorts that implement tau positron emission tomography (PET) quantification approaches to predict Alzheimer's disease (AD) progression. Most quantification approaches have been evaluated in isolation; head‐to‐head comparisons are rare, as are studies linking approaches to clinical endpoints.
2. **Interpretation**: In this multi‐cohort early AD sample, the group‐level temporal‐meta region of interest (ROI) consistently ranked among the top target regions for detecting tau PET change and clinical progression. Other approaches, including individualized (connectivity‐informed or Braak stage‐based) target ROIs as well as tau spatial extent (TAU‐SPEX), demonstrated their potential as complementary quantification metrics.
3. **Future directions**: Future work should test group‐level and individualized approaches in clinical trial datasets to determine which are most sensitive to treatment‐related changes in tau and clinical response, and which perform best under multi‐site conditions with variable follow‐up times and scanner/site effects. This would translate our head‐to‐head comparison into an evidence‐based selection of tau PET quantification approaches for clinical trials.

## 2 METHODS

### 2.1 Study sample

We included individuals across the AD continuum from seven cohorts (the 18F‐AV‐1451‐A05 flortaucipir PET study [NCT02016560; A05], the placebo arm of the Anti‐Amyloid Treatment in Asymptomatic Alzheimer's trial [NCT02008357; A4], the Longitudinal Evaluation of Amyloid Risk and Neurodegeneration observational cohort [NCT02488720; LEARN, a companion cohort to A4], the memory clinic–based Amsterdam Dementia Cohort [ADC], the Alzheimer's Disease Neuroimaging Initiative [ADNI], the Alzheimer's Disease Neuroimaging Initiative Department of Defense [ADNI DOD], and the Harvard Aging Brain study [NIH‐P01AG036694; HABS]). Eligible participants were confirmed Aβ positive (Aβ+; via PET or cerebrospinal fluid) and were either cognitively unimpaired (CU) at baseline or had a baseline clinical diagnosis of mild cognitive impairment (MCI) or mild dementia (dementia diagnosis with Mini‐Mental State Examination [MMSE] ≥ 20). Individuals with MCI and mild dementia were grouped into a single early symptomatic AD category (cognitively impaired) in line with clinical trials for AD. We collected basic demographic and clinical information (i.e., age, sex, education, apolipoprotein E [*APOE*] ε4 status, and MMSE). Participants were required to have at least two [^18^F]flortaucipir tau PET scans to evaluate longitudinal tau PET change; their baseline scans were used for cross‐sectional associations. Longitudinal neuropsychological assessments and clinical follow‐up were included when available and were used to characterize cognitive decline and clinical progression, but their unavailability was not an exclusion criterion. For grouping, when date information was available, Aβ status and, except when it was the outcome variable, clinical diagnosis had to be assessed within 1 year of the outcome variable (i.e., baseline tau PET scan, neuropsychological assessment, or clinical diagnosis). When Aβ status or clinical diagnosis at a given time point was missing but could be inferred from earlier or later visits in the same individual, we derived it accordingly (e.g., dementia or Aβ+ if already established at an earlier visit; CU or Aβ negative [Aβ–] if still applicable at a later visit). For A4 and LEARN, clinical diagnosis at each visit was derived from the Clinical Dementia Rating (CDR; CDR = 0: CU, CDR = 0.5 or 1: MCI and mild dementia).^35^, ^36^

### 2.2 Neuroimaging acquisition and processing

All participants had a minimum of two [^18^F]flortaucipir tau PET scans acquired according to site‐specific imaging protocols. Before processing, all PET and magnetic resonance imaging (MRI) scans were visually inspected to exclude those with artifacts or insufficient image quality. Image processing and ROI extraction were carried out centrally for all cohorts. PET scan frames were motion corrected and averaged to generate a mean image per scan, which was then co‐registered to the corresponding structural T1‐weighted MRI. To obtain regional tau PET standardized uptake value ratios (SUVRs), first the cortical Schaefer atlas^37^ (200 ROIs; 17 network parcellation) was mapped onto each participant's T1‐weighted MRI using CAT12. Then, [^18^F]flortaucipir signal within these ROIs was extracted from the co‐registered tau PET images and normalized to signal in the inferior cerebellar gray matter. This was used for the regional and global tau PET quantification approaches. The derivation of the voxel‐wise spatial extent metric is described in section 2.3.2.

### 2.3 Tau‐PET quantification approaches

**FIGURE 1:**
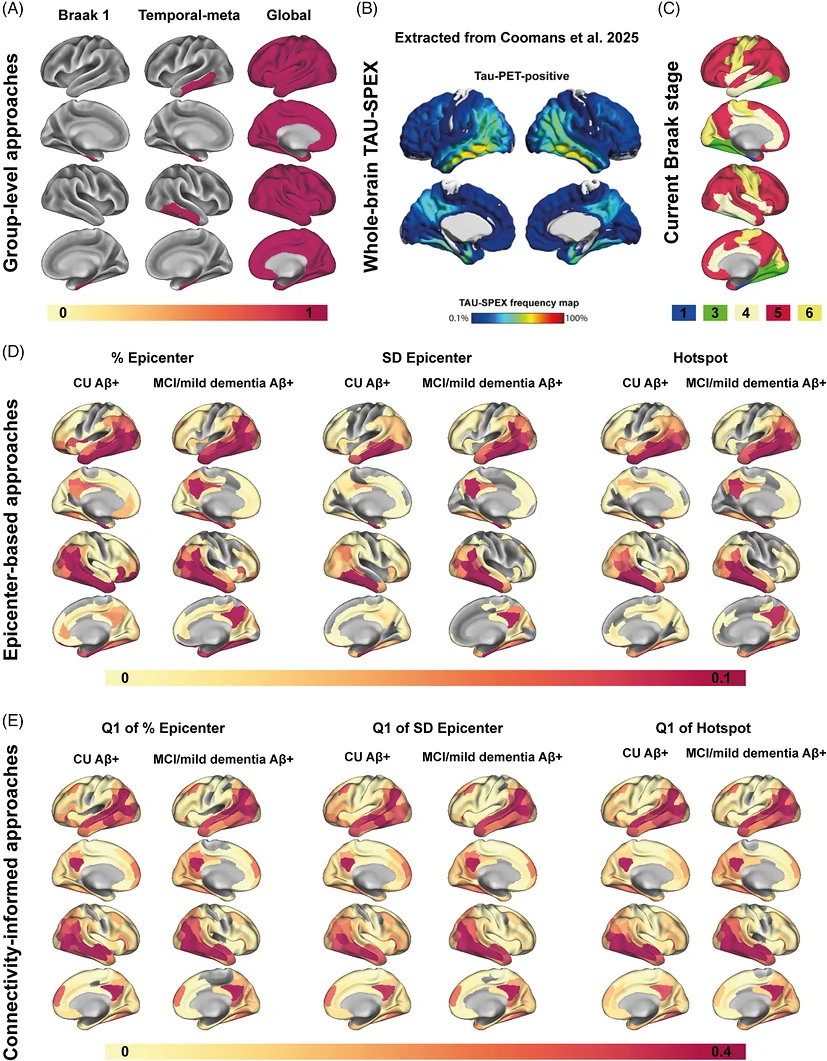
Overview of the different tau PET quantification approaches. We evaluated 11 tau PET quantification approaches: three group‐level approaches (Braak I, temporal‐meta, and global; A), a whole‐cortex metric of abnormal tau extent (TAU‐SPEX; B), the current Braak stage (C), three epicenter‐based approaches (D), and three connectivity‐informed approaches (E). All individualized approaches (C–E) were defined at baseline, and the same ROIs were used to extract SUVRs at each follow‐up visit. Braak I and temporal‐meta SUVRs reflect the mean across Schaefer ROIs overlapping the respective regions, and global SUVR reflects the mean across all 200 cortical Schaefer ROIs (A). TAU‐SPEX represents the proportion of cortical voxels above an abnormality threshold (SUVR ≥ 1.65^33^; B); the surface map shows the voxel‐wise frequency of tau positivity adapted from Coomans et al.^33^ Current Braak stage was assigned using Braak I and III‐VI composite ROIs (C). Epicenters were defined as: top 5% ROIs (% epicenter), ROIs exceeding mean + 2SD (SD epicenter), or the largest spatial cluster (“hotspot”) among top 5% ROIs. Group‐average epicenter/hotspot probabilities are shown in (D). Connectivity‐informed ROIs were derived using a normative functional connectivity matrix: at the subject level, for each seed definition (% epicenter, SD epicenter, hotspot), non‐seed ROIs were ranked by connectivity to the seed, and Q1 reflects the most strongly connected octile (≈ 12.5%); the surface renderings in (E) show group‐average Q1 probabilities. Aβ, amyloid beta; CU, cognitively unimpaired; MCI, mild cognitive impairment; PET, positron emission tomography; Q1, quantile 1; ROI, region of interest; SD, standard deviation; SUVR, standardized uptake value ratio; TAU‐SPEX, tau spatial extent.

We evaluated 11 literature‐derived tau PET approaches, selected to include both commonly used conventional ROIs and more recently proposed measures that have shown promise for capturing tau progression in AD. The selected approaches comprised three group‐level SUVR‐based ROIs, one individual‐level whole‐cortex metric of abnormal tau extent, and seven individualized SUVR‐based ROIs. For the individualized approaches, the set of Schaefer regions comprising each ROI was determined at baseline and then kept fixed across follow‐up visits, such that tau PET SUVR values at each visit were extracted from the same participant‐specific regions. An overview of all approaches can be found in Figure 1.

#### 2.3.1 Group‐level approaches

For the group‐level approaches, we included a Braak I ROI—representing the earliest tau stage measurable with PET^14^—derived by mapping the Schaefer 200 atlas onto a previously published Braak staging scheme.^8^, ^14^ We also extracted a temporal‐meta ROI, obtained by mapping the Schaefer 200 atlas onto a temporal‐meta ROI mask.^38^ Subsequently, we averaged the SUVR values for the Schaefer regions overlapping with the Braak I and temporal‐meta mask, respectively. In addition, we used global SUVR as an overall summary index, defined as the average of the ROI‐level tau PET SUVR values across all 200 cortical Schaefer ROIs.

#### 2.3.2 Whole‐brain spatial extent of abnormal tau

As a voxel‐based measure of tau pathology extent, we computed the proportion of cortical voxels with tau PET SUVR ≥ 1.65, a method previously referred to as TAU‐SPEX in the literature.^33^ This yielded a continuous whole‐brain spatial extent index representing the percentage of the cortex with elevated tau signal.

#### 2.3.3 Braak stage–based approach

For the individualized approaches, to assign a tau PET Braak stage to each individual, we first computed tau PET SUVRs for composite ROIs corresponding to standard Braak composites for stages I and III to VI (derived in the same way as described earlier for Braak I).^8^ For each Braak composite, we applied Gaussian mixture modeling (GMM; two‐component models) across all participants to estimate a sample‐level data‐driven intersection threshold between “low” and “high” tau distributions.^8^, ^30^ Using these group‐derived thresholds, for each subject and Braak composite, we then classified the stage as positive if the SUVR was greater than or equal to its corresponding GMM‐derived threshold.^8^ The current tau PET Braak stage was defined at the individual level as the highest Braak stage with a positive classification (i.e., positivity in earlier stages was not required). Subjects without any positive Braak stage were mapped to the Braak I composite to define the ROI for SUVR extraction. We then quantified the tau PET SUVR in the composite ROI corresponding to the individual's current tau PET Braak stage (see Figure S1 in supporting information for the current Braak stage distribution per diagnosis group at baseline).

#### 2.3.4 Epicenter‐based approaches

For each participant, we defined a tau PET epicenter by ranking all 200 ROIs by baseline tau PET SUVR and selecting the top 5% (i.e., the ROIs with the highest tau signal).^8^, ^28^, ^30^ The mean SUVR across these ROIs was used as the percent‐based (%) epicenter measure. In a second individualized epicenter approach, we computed the mean and standard deviation (SD) of baseline tau PET SUVR across all 200 ROIs for each participant and identified epicenter ROIs as those with SUVR values exceeding mean + 2SD within a given individual. The mean SUVR across these epicenter ROIs defined the SD‐based epicenter measure. If no ROI exceeded the threshold, the single ROI with the highest tau PET SUVR was selected as the epicenter. To capture clusters of neighboring regions with high tau burden as epicenters rather than potentially spatially scattered ROIs with high SUVRs, we added another, refined epicenter method: “hotspots.” For every individual, we clustered percent‐based epicenter ROIs based on their anatomical proximity. To that end, we computed a Euclidean distance matrix reflecting the geometric distance between the center of mass across ROIs. We then applied density‐based spatial clustering of applications with noise (DBSCAN), using an ε‐neighborhood radius of 15 mm (ε = 15 mm; ROIs within 15 mm Euclidean distance of each other are grouped together) and a minimum of two ROIs required to form a cluster (minPts = 2). Epicenter ROIs that did not belong to any cluster (noise points) were excluded, and the largest remaining cluster (i.e., the hotspot with the most ROIs) was used to derive the mean tau PET SUVR.

#### 2.3.5 Connectivity‐informed approaches

Using a previously established normative functional connectivity matrix^23^, ^39^ described in the Methods section in supporting information, we derived three connectivity‐informed tau PET measures anchored on (1) the percent‐based epicenter, (2) the SD‐based epicenter, and (3) the hotspot. For each of these seed definitions, and for each participant, we computed the mean functional connectivity–based distance from the seed ROIs to all other cortical ROIs. Target ROIs were then ranked and divided into eight octiles (i.e., quantile 1 [Q1] to quantile 8 [Q8]) based on connectivity strength, and Q1 was defined as the ROIs (≈ 12.5% of non‐epicenter ROIs) with the strongest connectivity to the respective seed. We then calculated the mean tau PET SUVR across Q1 ROIs for each seed, yielding three individualized measures that we refer to as Q1 percent‐based (%) epicenter, Q1 SD epicenter, and Q1 hotspot.

### 2.4 Tau PET change

We assessed longitudinal change in tau PET using linear mixed‐effects models including all scans per individual. For each ROI/metric, we fitted a separate model defined as: tau PET = β_0_ + β_1_ × time + b_0_i + b_1_i × time + ε, where time is the follow‐up time in years since the baseline tau PET scan, β_0_ and β_1_ are the fixed intercept and slope, b_0_i and b_1_i are the subject‐specific random intercept and slope, and ε is the residual error term. For each individual, the annual rate of change in tau PET was defined as the subject‐specific slope (β_1_ + b_1_i). Relative change (used for the analyses focused on quantifying tau PET change) was computed as (slope / baseline tau‐PET) × 100, yielding an annual percentage change in tau PET for each ROI/metric.

### 2.5 Neuropsychological testing

Longitudinal cognitive trajectories were evaluated with a cohort‐specific modified Preclinical Alzheimer Cognitive Composite (mPACC) score, designed to capture early AD‐related change across domains of episodic memory, executive function/processing speed, semantic fluency, and global cognition.^40^, ^41^ Neuropsychological assessments were included if performed after the baseline tau PET scan or within the preceding year. Baseline cognitive performance was defined using the first assessment occurring within ± 1 year of the baseline tau PET scan. When it was known that neuropsychological test scores and MMSE were not obtained on the same date, a maximum interval of 1 year between assessments was allowed. Within each cohort, test scores were *z* scored relative to that cohort's CU Aβ– reference group (A05 *n* = 119; A4 and LEARN [*z* scored together] *n* = 252; ADC *n* = 53; ADNI and ADNI DOD [*z* scored together] *n* = 163; HABS *n* = 288). For each available test (Table S1 in supporting information), *z* scores were calculated as z = (participant score – mean score CU Aβ–) / SD score CU Aβ–. Measures for which higher raw scores reflect worse performance were reverse‐scored so that higher *z* scores consistently indicated better performance. The mPACC5 score was computed as the mean of the available cohort‐specific *z* scored component tests, provided that at least two components were present and the available components were not restricted to two memory measures.

### 2.6 Clinical progression

Longitudinal clinical diagnosis information was included if obtained after the baseline tau PET scan or within the preceding year. Baseline clinical diagnosis was defined using the first assessment occurring within a maximum of ± 1 year from the baseline tau PET scan. Within the CU Aβ+ group, clinical progression was defined as progression from CU at baseline to MCI or mild dementia at the last available follow‐up. Within the MCI and mild dementia Aβ+ group, clinical progression was defined as progression to moderate/severe dementia (i.e., a dementia diagnosis with MMSE < 20) at the last available follow‐up. Clinical progression was coded as a binary indicator (1 = converter, 0 = non‐converter).

### 2.7 Statistical analyses

Group differences in baseline demographics were assessed using a Welch *t* test or Wilcoxon rank‐sum test for continuous variables and chi‐squared tests of independence for categorical variables. Participants with missing data were excluded per analysis (*n* = 2 education, *n* = 7 *APOE* ε4 carrier status).

All main analyses were performed within baseline diagnosis groups. To compare longitudinal annual percentage tau PET change between approaches, we computed paired (within‐subject) Cohen's *d* effect sizes for each approach versus the temporal‐meta ROI and for all pairwise comparisons across approaches, and tested whether the mean within‐subject difference between approaches differed from zero using two‐sided paired *t* tests. Multiple testing was controlled for using the Benjamini–Hochberg false discovery rate (FDR).

Sensitivity to longitudinal tau PET change was evaluated using linear mixed‐effects models for each approach, with follow‐up time (years) as the primary predictor, adjusting for baseline age, sex, and cohort, and including participant‐specific random intercepts and slopes. The fixed‐effect *t* value for time was extracted to summarize change. To assess the stability of *t* values, we performed participant‐level bootstrap resampling with replacement (1000 iterations; excluding unsuccessful fits). For each bootstrap sample, models were refit and *t* values were extracted; 95% confidence intervals (CIs) for the *t* values were then derived from the bootstrap distributions. Differences between approaches were assessed by computing, for each bootstrap iteration, pairwise differences in extracted *t* values. Two‐sided bootstrap *p* values were then computed to test whether these pairwise differences differed from zero; *p* values were FDR corrected.

Associations with longitudinal cognition (mPACC5 *z* scores; long format, including all available visits) were assessed using linear mixed‐effects models including time, the tau predictor (baseline or absolute rate of change), and their interaction (time **×** tau), adjusting for baseline age, sex, education, and cohort, with random intercepts and time slopes. We extracted the *t* value and *p* value for the interaction, and summarized model fit using marginal *R* ^2^. For baseline and longitudinal predictors separately, the approach with the highest marginal *R* ^2^ was compared against the others using 1000‐iteration participant‐level bootstrapping (with replacement) of *R* ^2^ differences, with 95% CI. A one‐sided *p* value was computed as the proportion of bootstrap iterations in which the *R* ^2^ difference was ≤ 0. Because comparisons were limited to the best‐performing approach versus each alternative, *p* values were interpreted without additional multiple comparisons correction.

Associations with clinical progression were assessed using logistic regression with progression status as the binary outcome and the tau quantification approach (baseline or absolute rate of change) as the main predictor, adjusting for baseline age, sex, and cohort. Covariate‐adjusted receiver operating characteristic (ROC) curves and areas under the ROC curve (AUC) were computed from model‐predicted probabilities; 95% CIs for AUCs were estimated using the DeLong method. For baseline and longitudinal predictors separately, the approach with the highest AUC was compared to the others using a DeLong test, reporting AUC differences with 95% CI.

Statistical significance was defined as two‐tailed *α *= 0.05. All statistical analyses were performed using R statistical software.

## 3 RESULTS

### 3.1 Participant characteristics

An overview of the study sample is provided in Table 1 (see Table S2 in supporting information for an overview of demographic and clinical information across cohorts). We included 335 CU Aβ+ individuals and 221 Aβ+ individuals with MCI or mild dementia who all had longitudinal tau PET data available. The CU Aβ+ group comprised relatively more females, had higher education, higher MMSE scores, and longer tau PET follow‐up time than the MCI and mild dementia Aβ+ group. Age and *APOE* ε4 carrier status did not differ between groups. Longitudinal neuropsychological testing data were available for 289 individuals in the CU Aβ+ group (follow‐up time: 4.31 ± 1.89 years) and 127 individuals in the MCI and mild dementia Aβ+ group (follow‐up time: 2.35 ± 1.74 years). Longitudinal clinical diagnosis data were available for 328 individuals in the CU Aβ+ group (follow‐up time: 5.07 ± 2.04 years) and 198 individuals in the MCI and mild dementia Aβ+ group (follow‐up time: 2.33 ± 1.84 years). The follow‐up time of longitudinal neuropsychological testing and clinical diagnosis data across cohorts is presented in Table S3 in supporting information.

**TABLE 1:** Demographic and clinical information across diagnosis groups.

	CU Aβ+	MCI and mild dementia Aβ+	
*N*	335	221	*p*
Age, yrs	72.93 (6.48)	73.59 (8.19)	0.316
Female	195 (58.2)	89 (40.3)	<0.001
Education, yrs	16.04 (2.82)	15.42 (2.97)	0.014
*APOE* ε4 carrier	196 (59.0)	136 (62.7)	0.446
MMSE	28.79 (1.35)	25.71 (2.87)	<0.001
Follow‐up time tau PET, yrs	3.72 (1.84)	1.90 (1.31)	<0.001

*Notes*: Values are mean (standard deviation) for continuous variables and *n* (%) for categorical variables. Group differences in baseline demographics were assessed using Welch *t* test or Wilcoxon rank‐sum test for continuous variables and chi‐squared tests of independence for categorical variables. Participants with missing data were excluded per analysis (*n* = 2 education, *n* = 7 *APOE* ε4 carrier status).

Abbreviations: Aβ, amyloid beta; *APOE*, apolipoprotein E; CU, cognitively unimpaired; MCI, mild cognitive impairment; MMSE, mini‐mental state examination; PET, positron emission tomography; yrs, years.

### 3.2 Quantification of longitudinal tau PET change

**FIGURE 2:**
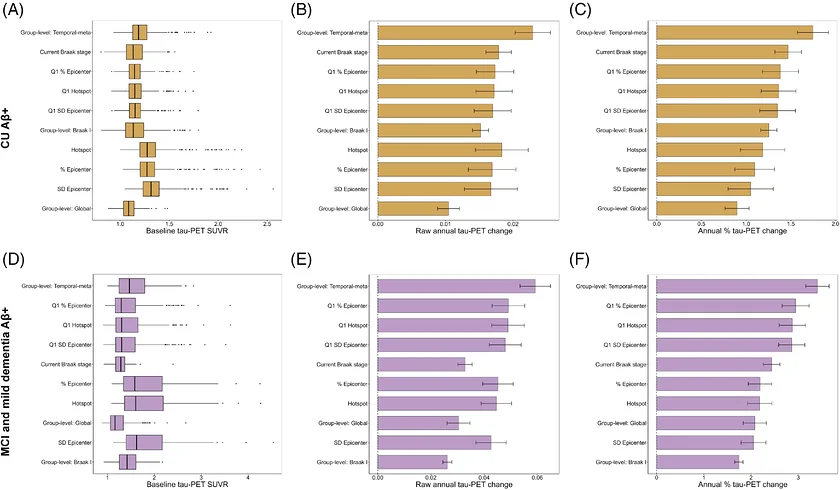
Baseline tau PET SUVR and annual raw and percentage tau PET change across approaches. For each approach, we fitted a separate linear mixed‐effects model defined as: tau PET = β_0_ + β_1_ × time + b_0_i + b_1_i × time + ε, where time is the follow‐up time in years since the baseline tau PET scan, β_0_ and β_1_ are the fixed intercept and slope, b_0_i and b_1_i are the subject‐specific random intercept and slope, and ε is the residual error term. The annual rate of change in tau PET (B, E) was defined as the subject‐specific slope (β_1_ + b_1_i). Relative change was computed as (slope / baseline tau‐PET) × 100, yielding an annual percentage change in tau PET for each approach (C, F), relative to the baseline tau PET SUVR (A, D). ROI order within each panel reflects the ranking of annual percentage tau PET change (C, F), applied consistently across all panels within each diagnosis group. Aβ, amyloid beta; CU, cognitively unimpaired; MCI, mild cognitive impairment; PET, positron emission tomography; Q1, quantile 1; ROI, region of interest; SD, standard deviation; SUVR, standardized uptake value ratio.

Our first objective was to assess differences in longitudinal percentage tau PET change across group‐level and individualized approaches. Figure 2 illustrates mean baseline tau PET SUVR, annual change in tau PET SUVR, and annual percentage change in tau PET SUVR across all approaches for the CU Aβ+ group (Figure 2A–C) and MCI and mild dementia Aβ+ group (Figure 2D–F); also see the Supplementary text and Figures S2 and S3 in supporting information.

**FIGURE 3:**
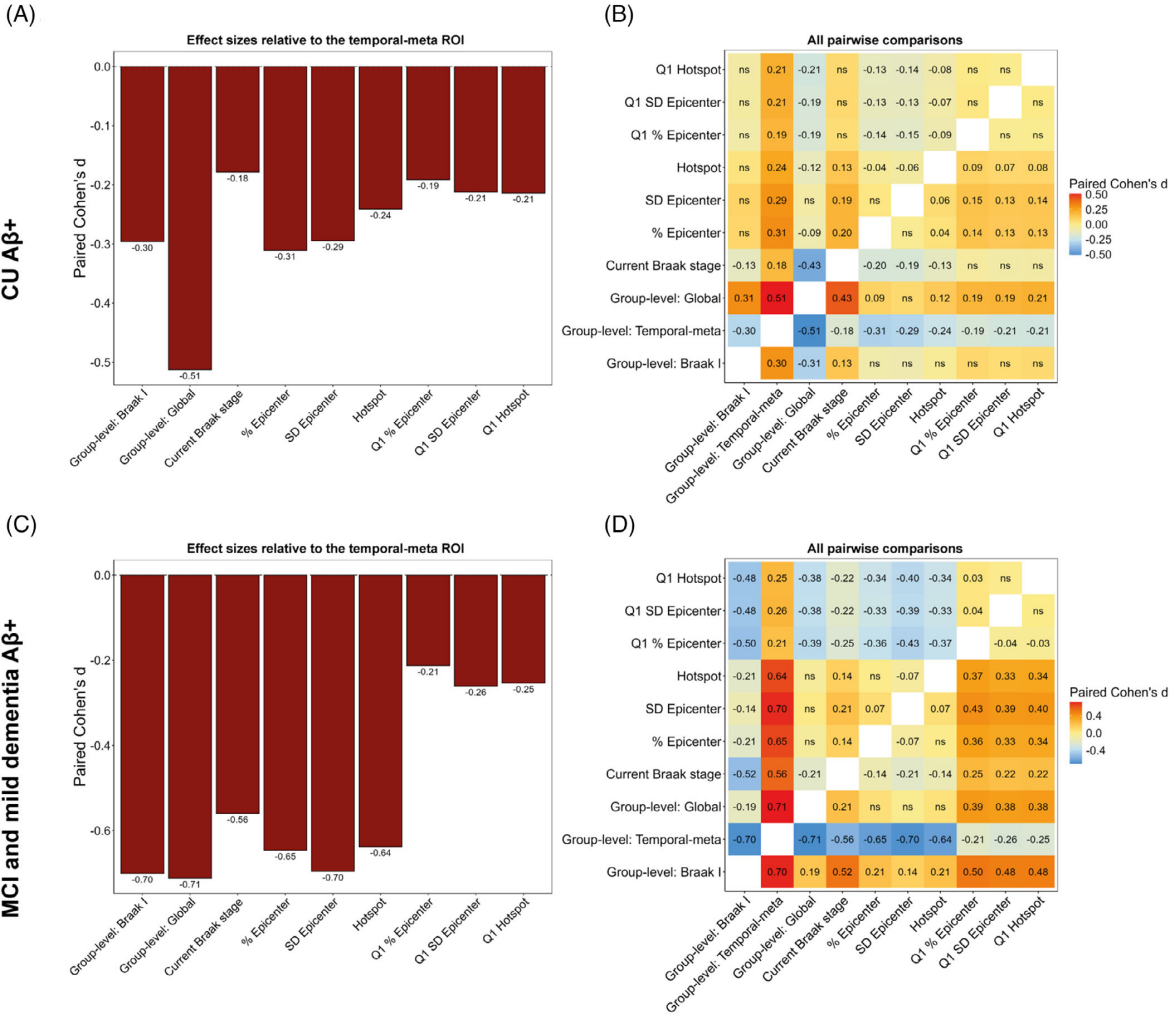
Comparing annual percentage tau PET change between approaches. To compare the longitudinal annual percentage tau PET change across approaches, we computed paired (within‐subject) Cohen's *d* effect sizes for each approach versus the temporal‐meta ROI (A, C) and for all pairwise comparisons across approaches (B, D). For inferential testing, two‐sided paired *t* tests were used to assess whether the mean within‐subject difference between approaches differed from zero. To account for multiple testing, *p* values were controlled using the Benjamini–Hochberg false discovery rate procedure. A, C, Bars represent paired Cohen's *d*, and red color indicates *p* < 0.001. B, D, Paired Cohen's *d* shown for significant comparisons, otherwise not significant. Aβ, amyloid beta; CU, cognitively unimpaired; MCI, mild cognitive impairment; PET, positron emission tomography; Q1, quantile 1; ROI, region of interest; SD, standard deviation.

Figure 3 demonstrates differences in percentage tau PET change between approaches. In both the CU Aβ+ group (Figure 3A) and MCI and mild dementia Aβ+ group (Figure 3C), the mean annual percentage change in tau PET was higher in the temporal‐meta ROI than in all other group‐level and individualized approaches (*p* < 0.001 for all comparisons). In the CU Aβ+ group, effect sizes for differences compared to the temporal‐meta ROI were small for most approaches (paired Cohen's *d* = –0.18 to –0.31) and moderate for the global approach (*d* = –0.51). In the MCI and mild dementia Aβ+ group, differences from the temporal‐meta ROI were larger overall, with small effect sizes for the Q1‐based approaches (*d* = –0.21 to –0.26) and moderate to large effect sizes for the remaining approaches (*d* = –0.56 to –0.71). Pairwise comparisons among the non‐temporal‐meta approaches further indicated that, in CU Aβ+ (Figure 3B), current Braak stage and the Q1‐based measures showed larger percentage tau PET change than the other approaches. In MCI and mild dementia Aβ+ (Figure 3D), the Q1‐based measures showed substantially larger percentage tau PET change than the remaining approaches.

These findings indicate that the temporal‐meta ROI consistently showed the greatest longitudinal tau PET change across AD disease stages, while Q1‐based approaches in both CU Aβ+ and MCI and mild dementia Aβ+, together with current Braak stage in CU Aβ+, ranked among the remaining methods with the largest percentage change.

### 3.3 Sensitivity to longitudinal tau PET change

**FIGURE 4:**
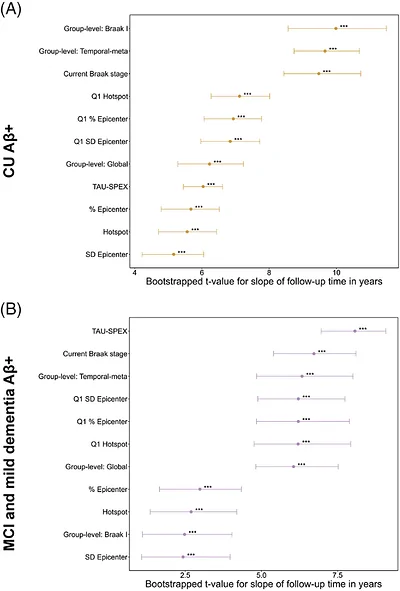
Sensitivity to tau PET change across approaches. To assess the sensitivity to detect longitudinal tau PET change across approaches, we modeled longitudinal tau PET trajectories per approach using linear mixed‐effects models with follow‐up time in years as the primary predictor, adjusting for baseline age, sex, and cohort, and including participant‐specific random intercepts and random slopes for follow‐up time. The fixed‐effect *t* value for follow‐up time was extracted for each approach as a summary measure of longitudinal change. To assess the stability of *t* values, we performed participant‐level bootstrap resampling with replacement (1000 iterations); iterations in which model fitting was unsuccessful (e.g., non‐convergence/errors) were excluded. For each bootstrap sample, models were refit and the *t* value for follow‐up time was extracted; 95% confidence intervals for the *t* values were obtained from the bootstrap distributions. Mean bootstrapped *t* values for the slope of follow‐up time in years are shown with confidence intervals for both CU Aβ+ (A) and MCI and mild dementia Aβ+ (B). Bootstrap 95% confidence intervals indicate some variation across approaches in the stability of the estimated time effects over resamples. This does not correspond one to one with the ranking based on mean bootstrapped *t* values, as the ranking reflects how strongly change was detected in the modeled data, whereas the confidence intervals reflect the stability of those estimates over resamples. *** indicates significance of the follow‐up time effect in the original linear mixed‐effects models (*p* < 0.001). Aβ, amyloid beta; CU, cognitively unimpaired; MCI, mild cognitive impairment; PET, positron emission tomography; Q1, quantile 1; ROI, region of interest; SD, standard deviation; TAU‐SPEX, tau spatial extent.

Our second objective was to compare tau PET quantification approaches in terms of their sensitivity to detect longitudinal tau PET change. Because this framework is scale independent, we were also able to include TAU‐SPEX in these comparisons alongside tau PET SUVR‐based methods. The results of these analyses are shown in Figure 4. In the CU Aβ+ group (Figure 4A), the group‐level Braak I (bootstrapped *t* = 9.986 [95% CI, 8.565–11.487]) and temporal‐meta (bootstrapped *t* = 9.663 [95% CI, 8.741–10.685]) ROIs, together with the individualized current Braak stage (bootstrapped *t* = 9.477 [95% CI, 8.439–10.727]), showed the highest bootstrapped *t* values for the follow‐up time slope, all significantly greater (i.e., *p* < 0.001) than those of the remaining approaches (Table S4 in supporting information). In the MCI and mild dementia Aβ+ group (Figure 4B), TAU‐SPEX showed the highest bootstrapped *t* value for the follow‐up time slope (bootstrapped *t* = 8.068 [95% CI, 6.956–9.081]), slightly higher than the current Braak stage (bootstrapped Δ*t* = 1.345 [95% CI, 0.278–2.445], *p* = 0.022) and temporal‐meta ROI (bootstrapped Δ*t* = 1.733 [95% CI, 0.371–2.968], *p* = 0.019), and substantially higher than the other approaches (all *p* < 0.001; Table S5 in supporting information). Current Braak stage, temporal‐meta, Q1‐based ROIs, and the global measure performed similarly, and were significantly more sensitive to longitudinal change than Braak I and the epicenter or hotspot approaches.

These results indicate that, in AD, at the CU stage, Braak I, temporal‐meta and current Braak stage had the largest and comparable sensitivity to detect longitudinal tau PET change, while at the MCI and mild dementia stage, TAU‐SPEX performed best, followed by current Braak stage, temporal‐meta, Q1‐based approaches and the global measure.

### 3.4 Baseline tau PET versus cognitive decline and clinical progression

**FIGURE 5:**
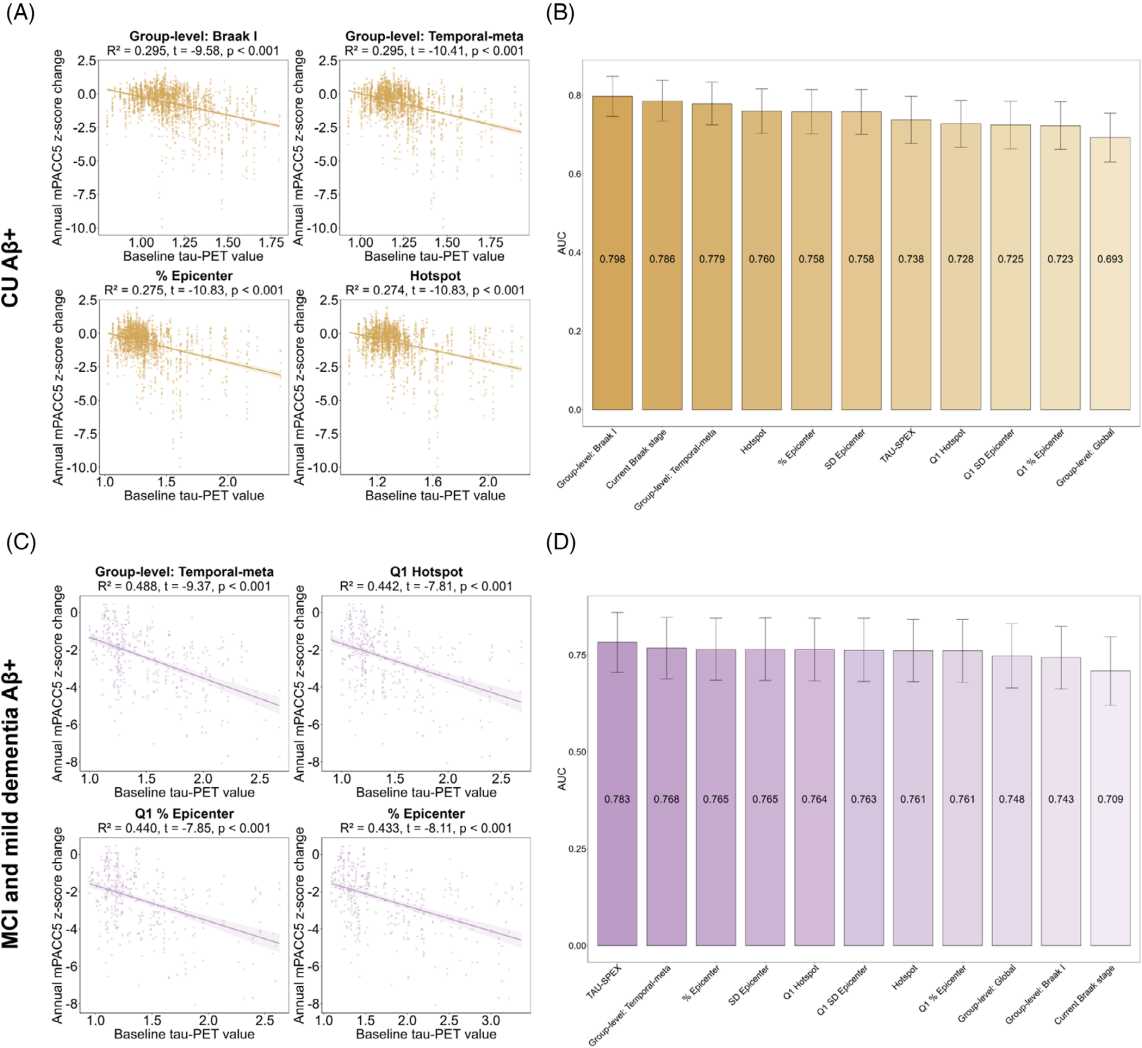
Associations of baseline tau PET with cognitive decline and clinical progression across approaches. To evaluate the association between each baseline tau PET approach and longitudinal cognitive decline (A, C), we fit separate linear mixed‐effects models with longitudinal mPACC5 composite *z* scores as the outcome. Models included follow‐up time (years), the tau predictor, and their interaction (time × tau), adjusting for baseline age, sex, education, and cohort, with participant‐specific random intercepts and random slopes for follow‐up time. The *t* value and *p* value for the time × tau interaction were extracted for each approach, and model fit was summarized using marginal *R* ^2^. Only the four approaches with the largest *R* ^2^ are shown. To assess the association between each baseline tau PET approach and clinical progression (B, D), we fit separate logistic regression models with progression status as the binary outcome (CU to MCI/mild dementia or MCI/mild dementia to moderate/severe dementia) and the tau PET approach as the main predictor, adjusting for baseline age, sex, and cohort. Model‐predicted probabilities were used to derive covariate‐adjusted ROC curves and AUCs; AUCs across approaches are presented. Aβ, amyloid beta; AUC, area under the curve; CU, cognitively unimpaired; MCI, mild cognitive impairment; mPACC, modified preclinical alzheimer cognitive composite; PET, positron emission tomography; Q1, quantile 1; ROC, receiver operating characteristic; ROI, region of interest; SD, standard deviation deviation; TAU‐SPEX, tau spatial extent.

Our third objective was to assess associations of baseline tau PET quantification approaches with cognitive decline and clinical progression. As shown in Figure 5, within the CU Aβ+ group (Figure 5A), baseline tau PET in the group‐level Braak I ROI accounted for the largest proportion of variance in mPACC5 *z* score change (*R* ^2^ = 0.295, *t* = –9.58, *p* < 0.001). Bootstrap analyses indicated that this *R* ^2^ was significantly higher than that of the group‐level global tau PET measure (bootstrapped Δ*R* ^2^ = 0.138 [95% CI, 0.082–0.202], *p* < 0.001), the individualized Q1% epicenter (bootstrapped Δ*R* ^2^ = 0.071 [95% CI, 0.015–0.132], *p* < 0.01), Q1 SD epicenter (bootstrapped Δ*R* ^2^ = 0.066 [95% CI, 0.012–0.128], *p* < 0.01), Q1 hotspot (bootstrapped Δ*R* ^2^ = 0.053 [95% CI, −0.001 to 0.113], *p* = 0.028), and current Braak stage (bootstrapped Δ*R* ^2^ = 0.053 [95% CI, –0.003 to 0.114], *p* = 0.033) approaches (Table S6 in supporting information). Braak stage I did not differ significantly from the remaining approaches.

In MCI and mild dementia Aβ+ (Figure 5C), baseline tau PET in the group‐level temporal‐meta ROI accounted for the largest proportion of variance in mPACC5 *z* score change (*R* ^2 ^= 0.488, *t* = –9.37, *p* < 0.001). Bootstrapping indicated that this *R* ^2^ was significantly higher than that of all other approaches, except for the Q1 hotspot and Q1% epicenter measures (Table S7 in supporting information).

Regarding clinical progression, in the CU Aβ+ group (Figure 5B), the group‐level Braak I ROI had the highest AUC for predicting progression to MCI and mild dementia (AUC = 0.798 [95% CI, 0.747–0.848]). As shown in Table S8 in supporting information, DeLong tests demonstrated that this AUC was significantly higher than that of the group‐level global approach (ΔAUC = 0.105 [95% CI, 0.056–0.153], *p* < 0.001), individualized Q1% epicenter (ΔAUC = 0.075 [95% CI, 0.027–0.122], *p* < 0.01), Q1 SD epicenter (ΔAUC = 0.073 [95% CI, 0.027–0.119], *p* < 0.01), and Q1 hotspot (ΔAUC = 0.070 [95% CI, 0.024–0.116], *p* < 0.01) approaches, and than that of TAU‐SPEX (ΔAUC = 0.060 [95% CI, 0.004–0.115], *p* = 0.036). The AUCs for the other approaches were statistically comparable.

In the MCI and mild dementia Aβ+ group (Figure 5D), TAU‐SPEX had the highest AUC for predicting progression to moderate/severe dementia (AUC = 0.783 [95% CI, 0.706–0.860]). However, DeLong tests indicated that this AUC was not significantly different from the other approaches (Table S9 in supporting information), except compared to the group‐level global tau PET measure (ΔAUC = 0.035 [95% CI, 0.008–0.063], *p* = 0.013).

These results indicate that, in CU Aβ+, baseline Braak I, temporal‐meta, epicenter/hotspot, and TAU‐SPEX measures performed comparably in predicting cognitive decline, whereas in MCI and mild dementia Aβ+, the temporal‐meta, Q1 hotspot, and Q1% epicenter measures were among the top‐performing predictors. For clinical progression, in CU Aβ+, Braak I, temporal‐meta, current Braak stage, and epicenter/hotspot approaches ranked among the strongest predictors, whereas in MCI and mild dementia Aβ+, all approaches performed similarly, except for the weaker performing global measure.

### 3.5 Longitudinal tau PET versus cognitive decline and clinical progression

**FIGURE 6:**
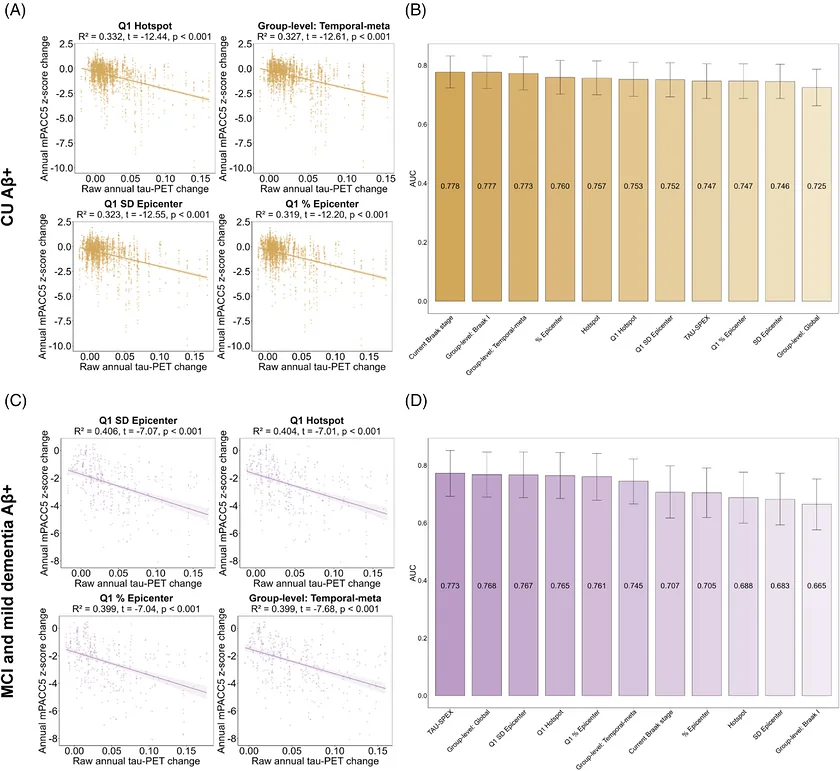
Associations of longitudinal tau PET change with cognitive decline and clinical progression across approaches. To evaluate the association between each longitudinal tau PET approach and longitudinal cognitive decline (A, C), we fit separate linear mixed‐effects models with mPACC5 composite *z* scores in long format as the outcome. Models included follow‐up time (years), the tau predictor (absolute rate of change), and their interaction (time × tau), adjusting for baseline age, sex, education, and cohort, with participant‐specific random intercepts and random slopes for follow‐up time. The *t* value and *p* value for the time × tau interaction were extracted for each approach, and model fit was summarized using marginal *R* ^2^. Only the four approaches with the largest *R* ^2^ are shown. To assess the association between each longitudinal tau PET approach and clinical progression (B, D), we fit separate logistic regression models with progression status as the binary outcome (CU to MCI/mild dementia or MCI/mild dementia to moderate/severe dementia) and the tau PET approach (absolute rate of change) as the main predictor, adjusting for baseline age, sex, and cohort. Model‐predicted probabilities were used to derive covariate‐adjusted ROC curves and AUCs; AUCs across approaches are presented. Aβ, amyloid beta; AUC, area under the curve; CU, cognitively unimpaired; MCI, mild cognitive impairment; mPACC, modified preclinical alzheimer cognitive composite; PET, positron emission tomography; Q1, quantile 1; ROC, receiver operating characteristic; ROI, region of interest; SD, standard deviation deviation; TAU‐SPEX, tau spatial extent.

Our last objective was to assess associations of tau PET change quantification approaches with cognitive decline and clinical progression. As shown in Figure 6, in the CU Aβ+ group (Figure 6A), tau PET change in the individualized Q1 hotspot approach accounted for the largest proportion of variance in mPACC5 *z* score change (*R* ^2^ = 0.332, *t* = –12.44, *p* < 0.001). Bootstrap analyses indicated that this *R* ^2^ was significantly higher than that of almost all other approaches, except for the group‐level temporal‐meta ROI, individualized Q1 SD epicenter approach, and TAU‐SPEX (Table S10 in supporting information).

In MCI and mild dementia Aβ+ (Figure 6C), tau PET change in the Q1 SD epicenter accounted for the largest proportion of variance in mPACC5 *z* score change (*R* ^2 ^= 0.406, *t* = –7.07, *p* < 0.001). Bootstrapping indicated that this *R* ^2^ was significantly higher than that of the individualized current Braak stage (bootstrapped Δ*R* ^2^ = 0.159 [95% CI, 0.029–0.234], *p* < 0.01) and SD epicenter (bootstrapped Δ*R* ^2^ = 0.150 [95% CI, 0.042–0.191], *p* < 0.001), the group‐level Braak I ROI (bootstrapped Δ*R* ^2^ = 0.144 [95% CI, 0.025–0.200], *p* < 0.01), as well as the individualized hotspot (bootstrapped Δ*R* ^2^ = 0.143 [95% CI, 0.033–0.175], *p* < 0.01) and % epicenter (bootstrapped Δ*R* ^2^ = 0.126 [95% CI, 0.028–0.157], *p* < 0.01; Table S11 in supporting information). The Q1 SD epicenter did not differ from the other approaches.

Regarding clinical progression, in CU Aβ+ (Figure 6B), the individualized current Braak stage had the highest AUC for predicting progression to MCI or mild dementia (AUC = 0.778 [95% CI, 0.724–0.832]). However, as shown in Table S12 in supporting information, DeLong tests indicated that this AUC was not significantly different from the AUC of the other approaches, except compared to the group‐level global measure (ΔAUC = 0.053 [95% CI, 0.011–0.095], *p* = 0.013).

In MCI and mild dementia Aβ+ (Figure 6D), TAU‐SPEX had the highest AUC for predicting progression to moderate/severe dementia (AUC = 0.773 [95% CI, 0.693–0.852]). DeLong tests demonstrated that this AUC was significantly higher than that of the group‐level Braak I ROI (ΔAUC = 0.108 [95% CI, 0.036–0.180], *p* < 0.01) and individualized SD epicenter (ΔAUC = 0.090 [95% CI, 0.027–0.153], *p* < 0.01), hotspot (ΔAUC = 0.085 [95% CI, 0.023–0.148], *p* < 0.01), and % epicenter (ΔAUC = 0.068 [95% CI, 0.013–0.123], *p* = 0.016) approaches (Table S13 in supporting information). The AUCs for the other approaches did not differ statistically.

These results indicate that, in CU Aβ+, longitudinal tau PET change measured using the Q1 hotspot, Q1 SD epicenter, TAU‐SPEX, and the temporal‐meta approaches performed similarly in predicting cognitive decline, whereas in MCI and mild dementia Aβ+, all Q1‐based approaches, TAU‐SPEX, the temporal‐meta ROI, and the global measure ranked among the strongest predictors. For clinical progression, in CU Aβ+, all approaches performed comparably except for the weaker performing global measure, whereas in MCI and mild dementia Aβ+, TAU‐SPEX, the global measure, Q1‐based approaches, the temporal‐meta ROI, and the current Braak stage showed similarly strong performance.

## 4 DISCUSSION

The main aim of this study was to determine whether individualized tau PET quantification approaches are more effective at capturing biological and clinical AD progression than conventional group‐level approaches. To address this, we performed a multi‐cohort longitudinal [^18^F]flortaucipir tau PET study, including 335 individuals with preclinical AD and 221 individuals with early symptomatic AD. Contrary to our hypothesis, the group‐level temporal‐meta ROI emerged as the best‐performing approach across disease stages and use cases, showing the largest longitudinal percentage tau PET change, high sensitivity to detect change (i.e., it clearly and reliably detected tau PET change over time), and strong associations with cognitive decline and clinical progression. Although several other approaches were non‐inferior in specific analyses, temporal‐meta remained one of the top performers. Considering its simplicity and consistently strong performance, the temporal‐meta ROI appears well suited as a tau PET endpoint in clinical trials. The added value of individualized metrics and the specific contexts in which they might be preferable require further investigation.

Although we did not expect the temporal‐meta ROI to outperform individualized ROIs, our observation that tau PET quantification in the temporal‐meta ROI provides robust longitudinal signal and pronounced clinical associations does align with prior work. Specifically, it has been well established that the temporal‐meta ROI can accurately capture progressive within‐person accumulation of tau over time, and that it has strong associations with cognitive decline.^11^, ^13^, ^42^, ^43^ Beyond temporal‐meta, other approaches also showed value and, except for percentage tau PET change, often performed comparably to it across use cases. In CU Aβ+, current Braak stage and Q1‐based approaches ranked among the strongest alternative methods for percentage tau PET change. Braak I and current Braak stage stood out for sensitivity to longitudinal tau PET change. For predicting cognitive decline, Braak I and epicenter/hotspot approaches performed well when baseline tau PET was used, and Q1‐based approaches when using longitudinal tau PET change. For predicting clinical progression, Braak I, current Braak stage, and epicenter/hotspot approaches performed well at baseline, while longitudinal approaches performed largely equally. In MCI and mild dementia Aβ+, Q1‐based approaches ranked among the strongest alternative methods for percentage tau PET change. TAU‐SPEX stood out for sensitivity to change. Q1‐based approaches were among the stronger predictors of cognitive decline for both baseline and longitudinal tau PET. For predicting clinical progression, baseline tau PET approaches performed largely similarly; longitudinally, especially TAU‐SPEX, the global measure, and Q1‐based approaches performed well.

The promising performance of current Braak stage, TAU‐SPEX, and Q1‐based ROIs for capturing biological and clinical disease progression is congruent with previous research. A recent head‐to‐head comparison of tau PET quantification approaches by Leuzy et al. showed that a data‐driven stage (DDS) method—similar to our current Braak stage approach, although based on data‐driven (rather than Braak‐based) stage composites—yielded the highest tau PET change, suggesting that quantifying tau load according to an individual's current pathological stage is relevant.^25^ Furthermore, TAU‐SPEX results align with prior work indicating that TAU‐SPEX can capture tau progression across the AD continuum and is associated with cognitive performance.^33^, ^44^, ^45^, ^46^ Finally, the findings on Q1 approaches are in line with studies showing that tau progresses through the brain from local epicenters in a connectivity‐dependent manner, leading to neurodegeneration and clinical symptoms that mirror the location of pathology.^8^, ^17^, ^23^, ^28^ In that context, this may also explain why the Q1‐based approaches showed stronger longitudinal performance than the epicenter/hotspot approaches, as the latter may already have been at a relatively more advanced stage of tau burden at baseline and therefore less dynamic for capturing subsequent change.^8^, ^23^ Our finding that especially baseline epicenter/hotspot tau PET load appeared relevant for predicting clinical progression is consistent with this. Overall, these findings suggest that while the temporal‐meta ROI remained the most robust approach, current Braak stage, TAU‐SPEX, and Q1‐based methods also emerged as promising tau PET–based endpoints in a stage‐ and use case–dependent manner. Specifically, current Braak stage was particularly informative in preclinical AD, TAU‐SPEX in early symptomatic AD, and Q1‐based measures in both disease stages, though with stronger performance in early symptomatic AD. The stronger performance of TAU‐SPEX in early symptomatic AD is likely explained by the relatively conservative voxel‐level tau PET positivity threshold (SUVR ≥ 1.65), which requires presence of substantial tau burden before TAU‐SPEX signal is detected. In contrast, region‐level extent measures based on data‐driven thresholds, such as tau magnitude and extent (Tau‐MaX),^47^ may be more sensitive to subtle early tau accumulation.

Interestingly, ROI rankings in Leuzy et al. differed from ours, with individualized ROIs outperforming the temporal‐meta ROI.^25^ Several methodological differences may contribute to this discrepancy. First, clinical grouping differed between studies, as Leuzy et al.^25^ analyzed MCI and AD dementia separately, whereas we combined MCI and mild AD dementia into a single early symptomatic group. Consequently, our approach may dilute stage‐specific changes in regional tau progression and thereby cause larger composites like temporal‐meta, which capture change across a wider symptomatic range,^48^ to be ranked higher. Differences in sample composition may also help explain why the maximum annual percentage change across groups was higher in Leuzy et al.^25^ (6.69%–10.74%) than in our study (1.75%–3.41%). Of note, the “early symptomatic AD” grouping we used is consistent with clinical trials that categorize participants with MCI and mild dementia into a single “early AD” entity.^5^, ^6^ Second, tracer differences may also play a role, as Leuzy et al.^25^ primarily analyzed a [^18^F]RO948 dataset, whereas our study used [^18^F]flortaucipir. Given differences in signal‐to‐noise ratios and binding profiles across tracers,^49^ this may have influenced ROI performance. Third, tau PET follow‐up was longer and more variable in our study and often included more than two tau PET scans, whereas Leuzy et al.^25^ included exactly two scans per participant, causing differences in how change was defined. Fourth, heterogeneity in scanners and acquisition protocols in multi‐cohort studies^50^, ^51^ may favor broader composite ROIs such as temporal‐meta, as they reduce measurement noise while still capturing disease progression, outweighing the disadvantage of averaging over regions with little or no change.^52^, ^53^ This consideration is relevant for clinical trial designs, as these are usually multi‐center and might have considerable variability between sites.^5^, ^6^ Last, differences in analytic framework should also be noted, as Leuzy et al.^25^ compared individualized ROIs against the best‐performing group‐level ROI using bootstrapping, whereas we compared all approaches using paired effect sizes (i.e., Cohen's *d*).

Our findings have practical implications for tau PET endpoint selection in AD trials. In a heterogeneous multi‐cohort setting, the temporal‐meta ROI provided the most consistently strong performance in capturing percentage change, sensitivity to change, and clinical associations across disease stages. This is pertinent to trial design as tau PET endpoints with greater mean longitudinal change relative to variability have a higher likelihood (i.e., greater power) of detecting treatment‐related slowing of tau accumulation.^11^, ^25^, ^54^ Additionally, high sensitivity to detect longitudinal tau PET change reflects the precision with which an approach can estimate group‐level change over time, accounting for within‐ and between‐subject heterogeneity.^11^, ^25^, ^54^, ^55^, ^56^ Strong associations with cognitive decline and clinical progression add additional value for a tau PET metric, as this increases the prognostic potential of tau PET. Interestingly, in an anti‐tau randomized clinical trial (i.e., BIIB080) the largest reductions in tau PET load from baseline were observed in a temporal composite region,^57^ demonstrating the relevance of composite temporal ROIs for clinical trials in AD. In summary, these results support the use of a group‐level temporal‐meta ROI as a pragmatic biomarker endpoint in multi‐center clinical trials to track overall disease progression, while the future of individualized measures may lie in personalized medicine strategies. Importantly, however, these findings are based on a general AD population using [^18^F]flortaucipir PET, and their applicability to atypical AD phenotypes or other tau PET tracers remains to be established.

Key strengths of this study are the head‐to‐head comparison of several common tau PET quantification methods across multiple outcomes relevant for clinical trial endpoint selection, the large sample size, inclusion of participants from preclinical AD to early symptomatic disease, the availability of more than two tau PET scans for many individuals, and the multi‐center design. The study integrates biological change, endpoint performance, and clinical relevance across disease stages. Importantly, beyond traditional SUVR‐based methods, we included TAU‐SPEX, a voxel‐based measure of abnormal tau extent,^33^ which provides an alternative way to measure tau progression that is agnostic to tau PET spreading patterns.

Several limitations should also be considered. First, the multi‐cohort design inevitably introduced heterogeneity, for example, in terms of recruitment strategies, scanner types, acquisition protocols, pre‐processing methodology, the availability of cognitive tests and clinical follow‐up, and the ascertainment of Aβ status. Second, tau PET has limited spatial resolution and is susceptible to partial‐volume effects and non‐specific binding, which could affect regional estimates of longitudinal change, particularly in small or anatomically complex regions.^58^, ^59^ Third, ROI extraction was performed using a cortical Schaefer 200 parcellation, which excludes subcortical structures^37^ and therefore did not allow us to assess subcortical tau accumulation. Fourth, due to scale differences, TAU‐SPEX is not directly comparable to SUVR‐based quantification metrics, limiting inclusion in percentage change–based comparisons, although it could be evaluated for sensitivity to change and clinical associations.

In conclusion, in this multi‐center study including preclinical to early symptomatic AD with longitudinal [^18^F]flortaucipir PET, the temporal‐meta ROI showed the most consistent performance in terms of capturing longitudinal percentage tau change, sensitivity to tau change, and predicting cognitive decline and clinical progression, supporting its role as a pragmatic tau PET endpoint in heterogeneous trial‐like settings. At the same time, connectivity‐informed measures, TAU‐SPEX, and current Braak stage also performed strongly, and could therefore be considered additional markers of biological and clinical disease progression, particularly in more homogeneous cohorts or settings  aimed at optimizing subject‐level tau detection. Future work should further evaluate candidate endpoints in trial simulations to determine which measures are most sensitive to treatment‐related tau change under trial conditions.

## AUTHOR CONTRIBUTIONS

Hannah de Bruin, Colin Groot, Rik Ossenkoppele, and Nicolai Franzmeier (Amsterdam, Munich) contributed to writing this article. The rest of the co‐authors critically reviewed the article.

## CONFLICT OF INTEREST STATEMENT

C.G. is supported by a Dementia Fellowship grant from ZonMW (10510022110010) and an Alzheimer Nederland Early Career Grant (project number: WE.03‐2024‐06). E.vd.G. has performed contract research for Heuron Inc., AC Immune, and Roche. She has a consultancy agreement with IXICO and Life Molecular Imaging for reading PET scans. All funding is paid to her institution. S.S., V.K., and D.O.S. are full‐time employees and minor shareholders of Eli Lilly and Company. Y.A.L.P. has received funding from the Dutch Brain Foundation, ZonMW, now, and the Mooiste Contact Fonds (both paid to her institution). E.M.C. is supported by an Alzheimer Nederland Early Career Grant (project number: WE.03‐2024‐03). R.O. is currently a full‐time employee of Eli Lilly and Company. His contribution to the work presented in this manuscript was performed as an employee of Amsterdam University Medical Centers. R.O. has received research funding/support from European Research Council, ZonMw, NWO, National Institute of Health, Alzheimer Association, Alzheimer Nederland, Stichting Dioraphte, Cure Alzheimer's fund, Health Holland, ERA PerMed, Alzheimerfonden, Hjarnfonden, Avid Radiopharmaceuticals, Janssen Research & Development, Roche, Quanterix, and Optina Diagnostics; has given lectures in symposia sponsored by GE Healthcare; received speaker fees from Springer; and was an advisory board/steering committee member for Asceneuron, Biogen, Johnson & Johnson, and Bristol Myers Squibb. All the aforementioned have been paid to Amsterdam University Medical Centers. N.F. has received funding from the Alzheimer's Association, Bright Focus Foundation, Alzheimer Forschung Initiative, Schick Foundation, Avid Radiopharmaceuticals, and Legerlotz Foundation and has received speaker or consulting honoraria from Eisai, Life Molecular Imaging, GE Healthcare, Biogen, and MSD. Author disclosures are available in the Supporting Information.

## CONSENT STATEMENT

All study procedures were conducted in accordance with the Declaration of Helsinki, and ethical approval was obtained by local investigators for each cohort. Participants provided written informed consent prior to enrollment.

## Supporting information




Supporting Information

ICMJE Disclosure Form
