# Supplementary material for: Group‐level versus individualized approaches for capturing tau PET changes and clinical progression in Alzheimer's disease
Source: Alzheimers Dement. 2026 Aug 19;22(8):e71718. doi: 10.1002/alz.71718 (PMC13490831; doi:10.1002/alz.71718)
Supplement: Supplementary file 2 [file ALZ-22-e71718-s002.docx]

**Supplementary methods**

We used resting-state fMRI data from an independent ADNI cohort of 42 CU Aβ- participants to build a connectivity template that served as the backbone for modeling tau progression from epicenter-based individualized ROIs. Preprocessing began with slice-timing and motion correction, with all volumes realigned to the first acquisition. Each participant’s Echo planar imaging (EPI) series was then co-registered to their T1-weighted structural image. Tissue masks for gray matter, eroded white matter, and eroded CSF were derived from the T1 scan and mapped into EPI space using rigid-body transformation.

For denoising, we removed nuisance variance by regressing out signals from eroded white matter and CSF, along with six motion parameters. The data were subsequently detrended and band-pass filtered (0.01-0.08 Hz), with all steps performed in native EPI space. To reduce motion-related bias in connectivity estimates, we applied motion scrubbing: frames with framewise displacement >0.5 mm were removed together with one preceding and two subsequent volumes. After censoring, we retained participants with ≥5 minutes of usable resting-state data. Spatial smoothing was not applied to avoid inflating connectivity via signal spillover between adjacent regions. The processed fMRI time series were normalized to MNI space using CAT12-derived normalization parameters.

Next, we parcellated the data using the 200 ROI cortical Schaefer atlas and computed functional connectivity across all regions as Fisher z–transformed Pearson correlations between ROI time series (i.e., blood oxygen level-dependent [BOLD] fluctuations). Following established procedures, the connectivity matrix was density-thresholded to 30%, retaining the strongest positive edges, and then converted to a functional connectivity-based distance representation.^1^ This distance measure captures the shortest functional path between regions, such that nodes linked by stronger direct or indirect connections are considered closer, whereas weakly connected or disconnected regions are farther apart.

References

1	Franzmeier, N. *et al.* Patient-centered connectivity-based prediction of tau pathology spread in Alzheimer's disease. *Sci Adv* **6** (2020). <https://doi.org/10.1126/sciadv.abd1327>

**Supplementary text**

As shown in **Figure 2** (main text), longitudinally, the group-level temporal-meta ROI exhibited the highest mean annual percentage change in tau-PET in both diagnosis groups (1.75% [95% CI, 1.57 - 1.92] in the CU Aβ+ group; 3.41% [95% CI, 3.16 - 3.66] in the MCI and mild dementia Aβ+ group), followed by the individualized approaches based on current Braak stage and Q1 of the epicenter (%/SD) or hotspot. Individual subject-level annual tau-PET rates of change (**A**) and annual percentage change in tau-PET (**B**) across all SUVR-based approaches in both groups can be found in **Supplementary Figure 2**. **Supplementary Figure 3** demonstrates that all SUVR-based approaches showed strong positive correlations with each other in both the CU Aβ+ (**A**) and MCI and mild dementia Aβ+ (**B**) group.

**Supplementary Figure 1. Current Braak stage distribution per diagnosis group at baseline.** Bars show the percentage of individuals assigned to each current Braak stage at baseline in the CU Aβ+ (**A**) and MCI and mild dementia Aβ+ (**B**) groups. Values above each bar denote the percentage of individuals in that stage. Aβ = amyloid-β; CU = cognitively unimpaired; MCI = mild cognitive impairment.

**Supplementary Figure 2. Individual subject-level annual tau-PET rates of change across quantification approaches.** Each panel shows the distribution of subject-level annual tau-PET change for one of the ten tau quantification approaches, in CU Aβ+ and MCI and mild dementia Aβ+ individuals, and for both annual absolute tau-PET change (**A**) and annual percentage tau-PET change (**B**). Aβ = amyloid-β; CU = cognitively unimpaired; MCI = mild cognitive impairment; PET = positron emission tomography; Q1 = quantile 1; SD = standard deviation.

**Supplementary Figure 3. Spearman correlation matrices of longitudinal tau-PET change across approaches.** Spearman correlation coefficients between annual absolute rates of change for all SUVR-based tau-PET quantification approaches are shown separately for the CU Aβ+ (**A**) and MCI and mild dementia Aβ+ (**B**) groups. Color intensity reflects the strength and direction of the correlation. All correlations were statistically significant (*p* < 0.05). Q1 = quantile 1; SD = standard deviation.

| **Cohort** | **Episodic memory** | **Executive functioning/speed** | **Semantic memory** | **Global cognition** |
| --- | --- | --- | --- | --- |
| **A05** | Wechsler IR + DR | TMT B | Animal fluency | MMSE |
| **A4** | FCSRT Total | DSST | n/a | MMSE |
| **ADC** | RAVLT IR + DR | TMT B | Animal fluency | MMSE |
| **ADNI** | RAVLT IR + DR | TMT B | Animal fluency | MMSE |
| **ADNI DOD** | RAVLT IR + DR | TMT B | Animal fluency | MMSE |
| **HABS** | SRT IR + DR | TMT B | Animal fluency | MMSE |
| **LEARN** | FCSRT Total | DSST | n/a | MMSE |

**Supplementary Table 1: Neuropsychological test inclusion across cohorts.** In most cohorts, the modified Preclinical Alzheimer Cognitive Composite (mPACC5) score consisted of five tests. In A05, the composite included the Wechsler Immediate Recall, Wechsler Delayed Recall, TMT B, Category Fluency Animals, and MMSE. In ADC, ADNI, and ADNI DOD, the composite comprised RAVLT Immediate Recall, RAVLT Delayed Recall, TMT B, Category Fluency Animals, and MMSE. In HABS, the composite included SRT Immediate Recall, SRT Delayed Recall, TMT B, Category Fluency Animals, and MMSE. Due to limited test availability, the A4 and LEARN mPACC5 score consisted of three tests: FCSRT Total, DSST, and MMSE. A05 = 18F-AV-1451-A05 flortaucipir PET study; A4 = anti-amyloid treatment in asymptomatic Alzheimer’s trial; ADC = Amsterdam dementia cohort; ADNI = Alzheimer's disease neuroimaging initiative; ADNI DOD = Alzheimer's disease neuroimaging initiative department of defense; DR = delayed recall; DSST = digit symbol substitution test; FCSRT = free and cued selective reminding test; HABS = Harvard aging brain study; IR = immediate recall; LEARN = longitudinal evaluation of amyloid risk and neurodegeneration observational cohort; MMSE = mini-mental state examination; RAVLT = Rey auditory verbal learning test; SRT = selective reminding test; TMT = trail making test.

|  | **A05** | **A4** | **ADC** | **ADNI** | **ADNI DOD** | **HABS** | **LEARN** | ***p*** |
| --- | --- | --- | --- | --- | --- | --- | --- | --- |
| ***N*** | 97 | 161 | 45 | 197 | 23 | 25 | 8 |  |
| **Diagnosis**^a^ |  |  |  |  |  |  |  | <0.001 |
| CU Aβ+ | 3 (3.1) | 161 (100.0) | 18 (40.0) | 103 (52.3) | 17 (73.9) | 25 (100.0) | 8 (100.0) |  |
| MCI/mild dementia Aβ+ | 94 (96.9) | 0 (0.0) | 27 (60.0) | 94 (47.7) | 6 (26.1) | 0 (0.0) | 0 (0.0) |  |
| **Age, yrs**^b^ | 74.54 (8.52) | 71.74 (4.71) | 66.53 (7.10) | 75.09 (7.35) | 71.13 (5.28) | 77.70 (5.20) | 69.06 (6.57) | <0.001 |
| **Female**^c^ | 43 (44.3) | 95 (59.0) | 21 (46.7) | 103 (52.3) | 1 (4.3) | 14 (56.0) | 7 (87.5) | <0.001 |
| **Education, yrs**^d^ | 15.59 (2.76) | 16.19 (2.89) | 12.33 (2.68) | 16.37 (2.42) | 14.70 (2.74) | 16.28 (2.91) | 17.62 (2.67) | <0.001 |
| ***APOE*ε4 carrier** | 55 (56.7) | 96 (59.6) | 31 (75.6) | 120 (61.5) | 10 (45.5) | 15 (60.0) | 5 (62.5) | 0.346 |
| **MMSE**^e^ | 25.49 (2.77) | 28.60 (1.31) | 26.58 (2.78) | 27.67 (2.69) | 27.83 (2.81) | 29.16 (0.99) | 29.25 (1.04) | <0.001 |
| **Follow-up tau-PET, yrs**^f^ | 1.11 (0.38) | 4.43 (1.40) | 2.30 (0.79) | 3.23 (1.96) | 1.22 (0.51) | 2.06 (0.62) | 3.28 (1.33) | <0.001 |

**Supplementary Table 2: Demographic and clinical information across cohorts.** Values are mean (standard deviation) for continuous variables and *n* (%) for categorical variables. Group differences in baseline demographics were assessed using one-way ANOVA and Kruskal-Wallis test for continuous variables and Chi-squared tests of independence for categorical variables. For post hoc tests, we used Tukey’s Honestly Significant Difference (HSD) following ANOVA, Dunn’s test following Kruskal-Wallis, and Fisher’s exact tests following Chi-squared tests. Dunn’s test and Fisher’s exact tests were FDR-corrected. Participants with missing data were excluded per analysis (*n* = 2 education, *n* = 7 *APOE*ε4 carrier status). Superscripts denote significant post hoc pairwise differences between cohorts (*p* < 0.05): ᵃDiagnosis differed for all cohort pairs except A4–HABS, A4–LEARN, ADC–ADNI, ADNI–ADNI DOD, ADNI DOD–LEARN, and HABS–LEARN; ᵇAge differed for A05–A4, A05–ADC, A4–ADC, A4–ADNI, A4–HABS, ADC–ADNI, ADC–HABS, ADNI DOD–HABS, and HABS–LEARN; ᶜSex differed between ADNI DOD and all other cohorts (no other pairs differed); ᵈEducation differed between ADC and all other cohorts (no other pairs differed), which may imply that the standard transformation of Verhage to years of education in ADC may not be accurate; ᵉMMSE differed for all cohort pairs except A4–ADNI DOD, A4–HABS, A4–LEARN, ADNI–ADNI DOD, ADNI–LEARN, ADNI DOD–HABS, ADNI DOD–LEARN, and HABS–LEARN; ᶠFollow-up time differed for all cohort pairs except A05–ADNI DOD, A4–LEARN, ADC–ADNI DOD, ADC–HABS, ADC–LEARN, ADNI–LEARN, ADNI DOD–HABS, and HABS–LEARN. A05 = 18F-AV-1451-A05 flortaucipir PET study; A4 = anti-amyloid treatment in asymptomatic Alzheimer’s trial; Aβ = amyloid-β; ADC = Amsterdam dementia cohort; ADNI = Alzheimer's disease neuroimaging initiative; ADNI DOD = Alzheimer's disease neuroimaging initiative department of defense; APOE = apolipoprotein E; CU = cognitively unimpaired; HABS = Harvard aging brain study; LEARN = longitudinal evaluation of amyloid risk and neurodegeneration observational cohort; MCI = mild cognitive impairment; MMSE = mini-mental state examination; PET = positron emission tomography; yrs = years.

|  | **A05** | **A4** | **ADC** | **ADNI** | **ADNI DOD** | **HABS** | **LEARN** | ***p*** |
| --- | --- | --- | --- | --- | --- | --- | --- | --- |
| **Longitudinal neuropsychological testing data** | | | | | | | | |
| ***N*** | 57 | 161 | 42 | 102 | 21 | 25 | 8 |  |
| **Follow-up, yrs**^a^ | 1.40 (0.31) | 5.27 (1.38) | 4.58 (2.45) | 2.90 (1.43) | 1.32 (0.67) | 2.60 (0.73) | 4.48 (1.66) | <0.001 |
| **Longitudinal clinical diagnosis data** | | | | | | | | |
| ***N*** | 96 | 161 | 24 | 197 | 15 | 25 | 8 |  |
| **Follow-up, yrs**^b^ | 1.11 (0.38) | 5.93 (1.50) | 5.17 (2.37) | 4.14 (2.03) | 1.36 (0.82) | 2.60 (0.73) | 4.94 (1.84) | <0.001 |

**Supplementary Table 3. Longitudinal follow-up time for neuropsychological testing and clinical diagnosis data across cohorts.** Values are mean (standard deviation). Group differences were assessed using one-way ANOVA. For post hoc tests, we used Tukey’s Honestly Significant Difference (HSD). ^a^Follow-up time for neuropsychological testing data differed between all cohort pairs except A05–ADNI DOD, A4–ADC, A4–LEARN, ADC–LEARN, and ADNI–HABS (all other pairs *p* < 0.05). ^b^Follow-up time for clinical diagnosis data differed between all cohort pairs except A05–ADNI DOD, A4–ADC, A4–LEARN, ADC–ADNI, ADC–LEARN, ADNI–LEARN, and ADNI DOD–HABS (all other pairs *p* < 0.05). A05 = 18F-AV-1451-A05 flortaucipir PET study; A4 = anti-amyloid treatment in asymptomatic Alzheimer’s trial; ADC = Amsterdam dementia cohort; ADNI = Alzheimer's disease neuroimaging initiative; ADNI DOD = Alzheimer's disease neuroimaging initiative department of defense; HABS = Harvard aging brain study; LEARN = longitudinal evaluation of amyloid risk and neurodegeneration observational cohort; yrs = years.

| **ROI 1** | **ROI 2** | **Δ*t*** | **CI lower** | **CI upper** | ***p*** |
| --- | --- | --- | --- | --- | --- |
| Global | Braak I | -3.759 | -5.236 | -2.431 | <0.001 |
| Global | Temporal-meta | -3.436 | -4.076 | -2.827 | <0.001 |
| Global | TAU-SPEX | 0.192 | -0.583 | 1.047 | 0.662 |
| Global | % Epicenter | 0.555 | -0.030 | 1.208 | 0.080 |
| Global | Q1 % Epicenter | -0.706 | -1.054 | -0.338 | <0.001 |
| Global | Hotspot | 0.666 | -0.043 | 1.428 | 0.080 |
| Global | Q1 Hotspot | -0.892 | -1.261 | -0.497 | <0.001 |
| Global | SD Epicenter | 1.067 | 0.361 | 1.829 | <0.001 |
| Global | Q1 SD Epicenter | -0.614 | -0.943 | -0.237 | <0.001 |
| Global | Current Braak stage | -3.251 | -4.353 | -2.253 | <0.001 |
| Braak I | Temporal-meta | 0.323 | -0.887 | 1.625 | 0.654 |
| Braak I | TAU-SPEX | 3.951 | 2.590 | 5.487 | <0.001 |
| Braak I | % Epicenter | 4.314 | 3.023 | 5.854 | <0.001 |
| Braak I | Q1 % Epicenter | 3.054 | 1.716 | 4.578 | <0.001 |
| Braak I | Hotspot | 4.425 | 3.128 | 5.919 | <0.001 |
| Braak I | Q1 Hotspot | 2.867 | 1.548 | 4.347 | <0.001 |
| Braak I | SD Epicenter | 4.826 | 3.492 | 6.351 | <0.001 |
| Braak I | Q1 SD Epicenter | 3.145 | 1.755 | 4.657 | <0.001 |
| Braak I | Current Braak stage | 0.508 | -0.343 | 1.348 | 0.290 |
| Temporal-meta | TAU-SPEX | 3.628 | 2.846 | 4.474 | <0.001 |
| Temporal-meta | % Epicenter | 3.991 | 3.327 | 4.782 | <0.001 |
| Temporal-meta | Q1 % Epicenter | 2.730 | 2.183 | 3.291 | <0.001 |
| Temporal-meta | Hotspot | 4.102 | 3.350 | 4.956 | <0.001 |
| Temporal-meta | Q1 Hotspot | 2.544 | 2.004 | 3.071 | <0.001 |
| Temporal-meta | SD Epicenter | 4.503 | 3.743 | 5.416 | <0.001 |
| Temporal-meta | Q1 SD Epicenter | 2.822 | 2.278 | 3.393 | <0.001 |
| Temporal-meta | Current Braak stage | 0.185 | -0.744 | 1.046 | 0.662 |
| TAU-SPEX | % Epicenter | 0.363 | -0.280 | 1.063 | 0.299 |
| TAU-SPEX | Q1 % Epicenter | -0.897 | -1.592 | -0.255 | 0.006 |
| TAU-SPEX | Hotspot | 0.474 | -0.222 | 1.164 | 0.206 |
| TAU-SPEX | Q1 Hotspot | -1.084 | -1.774 | -0.432 | 0.003 |
| TAU-SPEX | SD Epicenter | 0.875 | 0.140 | 1.619 | 0.016 |
| TAU-SPEX | Q1 SD Epicenter | -0.806 | -1.517 | -0.129 | 0.028 |
| TAU-SPEX | Current Braak stage | -3.443 | -4.608 | -2.390 | <0.001 |
| % Epicenter | Q1 % Epicenter | -1.261 | -1.772 | -0.772 | <0.001 |
| % Epicenter | Hotspot | 0.111 | -0.174 | 0.395 | 0.478 |
| % Epicenter | Q1 Hotspot | -1.447 | -2.016 | -0.962 | <0.001 |
| % Epicenter | SD Epicenter | 0.512 | 0.217 | 0.823 | <0.001 |
| % Epicenter | Q1 SD Epicenter | -1.169 | -1.662 | -0.729 | <0.001 |
| % Epicenter | Current Braak stage | -3.806 | -4.992 | -2.779 | <0.001 |
| Q1 % Epicenter | Hotspot | 1.372 | 0.785 | 2.004 | <0.001 |
| Q1 % Epicenter | Q1 Hotspot | -0.186 | -0.374 | 0.002 | 0.064 |
| Q1 % Epicenter | SD Epicenter | 1.773 | 1.176 | 2.398 | <0.001 |
| Q1 % Epicenter | Q1 SD Epicenter | 0.091 | -0.100 | 0.296 | 0.388 |
| *table continues on the next page* | | | | | |
| *table continues* | | | | | |
| **ROI 1** | **ROI 2** | **Δ*t*** | **CI lower** | **CI upper** | ***p*** |
| Q1 % Epicenter | Current Braak stage | -2.545 | -3.609 | -1.549 | <0.001 |
| Hotspot | Q1 Hotspot | -1.558 | -2.215 | -1.002 | <0.001 |
| Hotspot | SD Epicenter | 0.401 | 0.045 | 0.749 | 0.045 |
| Hotspot | Q1 SD Epicenter | -1.280 | -1.917 | -0.744 | <0.001 |
| Hotspot | Current Braak stage | -3.917 | -5.107 | -2.905 | <0.001 |
| Q1 Hotspot | SD Epicenter | 1.959 | 1.362 | 2.619 | <0.001 |
| Q1 Hotspot | Q1 SD Epicenter | 0.278 | 0.061 | 0.497 | 0.013 |
| Q1 Hotspot | Current Braak stage | -2.359 | -3.427 | -1.379 | <0.001 |
| SD Epicenter | Q1 SD Epicenter | -1.681 | -2.245 | -1.128 | <0.001 |
| SD Epicenter | Current Braak stage | -4.318 | -5.560 | -3.250 | <0.001 |
| Q1 SD Epicenter | Current Braak stage | -2.637 | -3.718 | -1.629 | <0.001 |

**Supplementary Table 4: Comparing sensitivity to tau-PET change across approaches in CU Aβ+.** Bootstrapped *t*-values for the follow-up time slope (with longitudinal tau-PET as the outcome) were obtained from linear mixed-effects models as described in **Figure 4**. Differences between approaches in sensitivity to detect longitudinal tau-PET change were assessed by computing, across bootstrap iterations, the pairwise difference in extracted *t*-values. Two-sided bootstrap *p*-values were derived from the proportion of iterations in which the difference was above versus below zero, and 95% confidence intervals were obtained from the bootstrap distribution of differences; *p*-values were FDR-corrected. CI = confidence interval; Q1 = quantile 1; ROI = region of interest; SD = standard deviation; TAU-SPEX = tau spatial extent.

| **ROI 1** | **ROI 2** | **Δ*t*** | **CI lower** | **CI upper** | ***p*** |
| --- | --- | --- | --- | --- | --- |
| Global | Braak I | 3.577 | 2.211 | 5.027 | <0.001 |
| Global | Temporal-meta | -0.280 | -1.355 | 0.873 | 0.782 |
| Global | TAU-SPEX | -2.013 | -2.862 | -0.975 | <0.001 |
| Global | % Epicenter | 3.073 | 2.086 | 4.177 | <0.001 |
| Global | Q1 % Epicenter | -0.156 | -0.810 | 0.426 | 0.782 |
| Global | Hotspot | 3.361 | 2.281 | 4.582 | <0.001 |
| Global | Q1 Hotspot | -0.150 | -0.807 | 0.464 | 0.782 |
| Global | SD Epicenter | 3.626 | 2.606 | 4.740 | <0.001 |
| Global | Q1 SD Epicenter | -0.157 | -0.695 | 0.378 | 0.706 |
| Global | Current Braak stage | -0.668 | -1.758 | 0.431 | 0.356 |
| Braak I | Temporal-meta | -3.857 | -5.016 | -2.745 | <0.001 |
| Braak I | TAU-SPEX | -5.591 | -6.998 | -4.087 | <0.001 |
| Braak I | % Epicenter | -0.504 | -1.568 | 0.590 | 0.488 |
| Braak I | Q1 % Epicenter | -3.733 | -5.364 | -2.322 | <0.001 |
| Braak I | Hotspot | -0.216 | -1.300 | 0.892 | 0.802 |
| Braak I | Q1 Hotspot | -3.727 | -5.319 | -2.305 | <0.001 |
| Braak I | SD Epicenter | 0.048 | -1.073 | 1.202 | 0.942 |
| Braak I | Q1 SD Epicenter | -3.734 | -5.171 | -2.316 | <0.001 |
| Braak I | Current Braak stage | -4.246 | -5.558 | -2.906 | <0.001 |
| Temporal-meta | TAU-SPEX | -1.733 | -2.968 | -0.371 | 0.019 |
| Temporal-meta | % Epicenter | 3.353 | 2.484 | 4.468 | <0.001 |
| Temporal-meta | Q1 % Epicenter | 0.124 | -1.159 | 1.367 | 0.921 |
| Temporal-meta | Hotspot | 3.641 | 2.751 | 4.664 | <0.001 |
| Temporal-meta | Q1 Hotspot | 0.130 | -1.099 | 1.317 | 0.921 |
| Temporal-meta | SD Epicenter | 3.905 | 2.958 | 5.055 | <0.001 |
| Temporal-meta | Q1 SD Epicenter | 0.123 | -0.973 | 1.257 | 0.921 |
| Temporal-meta | Current Braak stage | -0.389 | -1.759 | 1.023 | 0.782 |
| TAU-SPEX | % Epicenter | 5.087 | 3.911 | 6.265 | <0.001 |
| TAU-SPEX | Q1 % Epicenter | 1.857 | 0.538 | 2.908 | <0.001 |
| TAU-SPEX | Hotspot | 5.374 | 4.090 | 6.600 | <0.001 |
| TAU-SPEX | Q1 Hotspot | 1.864 | 0.514 | 2.953 | <0.001 |
| TAU-SPEX | SD Epicenter | 5.639 | 4.419 | 6.852 | <0.001 |
| TAU-SPEX | Q1 SD Epicenter | 1.856 | 0.668 | 2.892 | <0.001 |
| TAU-SPEX | Current Braak stage | 1.345 | 0.278 | 2.445 | 0.022 |
| % Epicenter | Q1 % Epicenter | -3.229 | -4.308 | -2.355 | <0.001 |
| % Epicenter | Hotspot | 0.288 | -0.007 | 0.584 | 0.092 |
| % Epicenter | Q1 Hotspot | -3.223 | -4.318 | -2.376 | <0.001 |
| % Epicenter | SD Epicenter | 0.552 | 0.290 | 0.813 | <0.001 |
| % Epicenter | Q1 SD Epicenter | -3.231 | -4.142 | -2.412 | <0.001 |
| % Epicenter | Current Braak stage | -3.742 | -5.111 | -2.383 | <0.001 |
| Q1 % Epicenter | Hotspot | 3.517 | 2.528 | 4.671 | <0.001 |
| Q1 % Epicenter | Q1 Hotspot | 0.006 | -0.184 | 0.187 | 0.942 |
| Q1 % Epicenter | SD Epicenter | 3.781 | 2.936 | 4.874 | <0.001 |
| Q1 % Epicenter | Q1 SD Epicenter | -0.001 | -0.254 | 0.281 | 0.936 |
| *table continues on the next page* | | | | | |
| *table continues* | | | | | |
| **ROI 1** | **ROI 2** | **Δ*t*** | **CI lower** | **CI upper** | ***p*** |
| Q1 % Epicenter | Current Braak stage | -0.513 | -1.826 | 0.925 | 0.604 |
| Hotspot | Q1 Hotspot | -3.511 | -4.688 | -2.547 | <0.001 |
| Hotspot | SD Epicenter | 0.264 | -0.107 | 0.638 | 0.229 |
| Hotspot | Q1 SD Epicenter | -3.518 | -4.532 | -2.582 | <0.001 |
| Hotspot | Current Braak stage | -4.030 | -5.473 | -2.500 | <0.001 |
| Q1 Hotspot | SD Epicenter | 3.775 | 2.919 | 4.847 | <0.001 |
| Q1 Hotspot | Q1 SD Epicenter | -0.008 | -0.283 | 0.345 | 0.936 |
| Q1 Hotspot | Current Braak stage | -0.519 | -1.847 | 0.983 | 0.613 |
| SD Epicenter | Q1 SD Epicenter | -3.783 | -4.728 | -2.978 | <0.001 |
| SD Epicenter | Current Braak stage | -4.294 | -5.704 | -2.895 | <0.001 |
| Q1 SD Epicenter | Current Braak stage | -0.511 | -1.838 | 0.836 | 0.602 |

**Supplementary Table 5: Comparing sensitivity to tau-PET change across approaches in MCI and mild dementia Aβ+.** Bootstrapped *t*-values for the follow-up time slope (with longitudinal tau-PET as the outcome) were obtained from linear mixed-effects models as described in **Figure 4**. Differences between approaches in sensitivity to detect longitudinal tau-PET change were assessed by computing, across bootstrap iterations, the pairwise difference in extracted *t*-values. Two-sided bootstrap *p*-values were derived from the proportion of iterations in which the difference was above versus below zero, and 95% confidence intervals were obtained from the bootstrap distribution of differences; *p*-values were FDR-corrected. CI = confidence interval; Q1 = quantile 1; ROI = region of interest; SD = standard deviation; TAU-SPEX = tau spatial extent.

| **Best ROI** | **Comparator ROI** | **Δ*R^2^*** | **CI lower** | **CI upper** | ***p*** |
| --- | --- | --- | --- | --- | --- |
| Braak I | Global | 0.138 | 0.082 | 0.202 | <0.001 |
| Braak I | Temporal-meta | 0.000 | -0.058 | 0.060 | 0.500 |
| Braak I | TAU-SPEX | 0.042 | -0.042 | 0.115 | 0.139 |
| Braak I | % Epicenter | 0.019 | -0.037 | 0.092 | 0.249 |
| Braak I | Q1 % Epicenter | 0.071 | 0.015 | 0.132 | 0.005 |
| Braak I | Hotspot | 0.021 | -0.037 | 0.088 | 0.216 |
| Braak I | Q1 Hotspot | 0.053 | -0.001 | 0.113 | 0.028 |
| Braak I | SD Epicenter | 0.027 | -0.031 | 0.100 | 0.173 |
| Braak I | Q1 SD Epicenter | 0.066 | 0.012 | 0.128 | 0.003 |
| Braak I | Current Braak stage | 0.053 | -0.003 | 0.114 | 0.033 |

**Supplementary Table 6: Bootstrapped *R*^2^ comparisons between approaches for the association between baseline tau-PET and cognitive decline in CU Aβ+.** Marginal *R*^2^ values were obtained from the linear mixed-effects models relating baseline tau-PET to longitudinal mPACC5 change as described in **Figure 5**. The approach with the highest marginal *R*^2^ in the original sample was treated as the reference. Participant-level bootstrap resampling with replacement (1000 iterations; unsuccessful fits excluded) was used to refit the reference model and each comparator model within each bootstrap sample, and the pairwise difference in marginal *R*^2^ (reference − comparator) was computed per iteration. 95% confidence intervals were derived from the bootstrap distribution of *R*^2^ differences. One-sided bootstrap *p*-values were computed as the proportion of iterations in which the *R*^2^ difference was ≤0 (i.e., comparator performed at least as well as the reference). CI = confidence interval; Q1 = quantile 1; ROI = region of interest; SD = standard deviation; TAU-SPEX = tau spatial extent.

| **Best ROI** | **Comparator ROI** | **Δ*R^2^*** | **CI lower** | **CI upper** | ***p*** |
| --- | --- | --- | --- | --- | --- |
| Temporal-meta | Global | 0.091 | 0.018 | 0.117 | 0.001 |
| Temporal-meta | Braak I | 0.134 | 0.030 | 0.193 | 0.002 |
| Temporal-meta | TAU-SPEX | 0.080 | 0.007 | 0.133 | 0.017 |
| Temporal-meta | % Epicenter | 0.055 | -0.002 | 0.097 | 0.032 |
| Temporal-meta | Q1 % Epicenter | 0.048 | -0.015 | 0.101 | 0.057 |
| Temporal-meta | Hotspot | 0.070 | 0.010 | 0.108 | 0.010 |
| Temporal-meta | Q1 Hotspot | 0.047 | -0.019 | 0.089 | 0.098 |
| Temporal-meta | SD Epicenter | 0.060 | 0.004 | 0.098 | 0.021 |
| Temporal-meta | Q1 SD Epicenter | 0.059 | -0.013 | 0.115 | 0.046 |
| Temporal-meta | Current Braak stage | 0.244 | 0.074 | 0.328 | 0.001 |

**Supplementary Table 7: Bootstrapped *R*^2^ comparisons between approaches for the association between baseline tau-PET and cognitive decline in MCI and mild dementia Aβ+.** Marginal *R*^2^ values were obtained from the linear mixed-effects models relating baseline tau-PET to longitudinal mPACC5 change as described in **Figure 5**. The approach with the highest marginal *R*^2^ in the original sample was treated as the reference. Participant-level bootstrap resampling with replacement (1000 iterations; unsuccessful fits excluded) was used to refit the reference model and each comparator model within each bootstrap sample, and the pairwise difference in marginal *R*^2^ (reference − comparator) was computed per iteration. 95% confidence intervals were derived from the bootstrap distribution of *R*^2^ differences. One-sided bootstrap *p*-values were computed as the proportion of iterations in which the *R*^2^ difference was ≤0 (i.e., comparator performed at least as well as the reference). CI = confidence interval; Q1 = quantile 1; ROI = region of interest; SD = standard deviation; TAU-SPEX = tau spatial extent.

| **Best ROI** | **Comparator ROI** | **ΔAUC** | **CI lower** | **CI upper** | ***p*** |
| --- | --- | --- | --- | --- | --- |
| Braak I | Current Braak stage | 0.011 | -0.009 | 0.031 | 0.265 |
| Braak I | Temporal-meta | 0.019 | -0.015 | 0.052 | 0.267 |
| Braak I | Hotspot | 0.038 | -0.007 | 0.083 | 0.098 |
| Braak I | % Epicenter | 0.039 | -0.006 | 0.085 | 0.091 |
| Braak I | SD Epicenter | 0.040 | -0.006 | 0.085 | 0.086 |
| Braak I | TAU-SPEX | 0.060 | 0.004 | 0.115 | 0.036 |
| Braak I | Q1 Hotspot | 0.070 | 0.024 | 0.116 | 0.003 |
| Braak I | Q1 SD Epicenter | 0.073 | 0.027 | 0.119 | 0.002 |
| Braak I | Q1 % Epicenter | 0.075 | 0.027 | 0.122 | 0.002 |
| Braak I | Global | 0.105 | 0.056 | 0.153 | <0.001 |

**Supplementary Table 8: Comparing AUCs for the association between baseline tau-PET and clinical progression across approaches in CU Aβ+.** For each baseline tau-PET approach, covariate-adjusted AUCs were computed from logistic regression model-predicted probabilities, as described in **Figure 5**. The approach with the highest AUC was identified as the reference and compared against each of the other approaches using DeLong’s test, reporting the AUC difference with 95% confidence intervals and providing two-sided *p*-values for each comparison. AUC = area under the curve; CI = confidence interval; Q1 = quantile 1; ROI = region of interest; SD = standard deviation; TAU-SPEX = tau spatial extent.

| **Best ROI** | **Comparator ROI** | **ΔAUC** | **CI lower** | **CI upper** | ***p*** |
| --- | --- | --- | --- | --- | --- |
| TAU-SPEX | Temporal-meta | 0.016 | -0.022 | 0.053 | 0.416 |
| TAU-SPEX | % Epicenter | 0.018 | -0.011 | 0.046 | 0.219 |
| TAU-SPEX | SD Epicenter | 0.018 | -0.008 | 0.044 | 0.165 |
| TAU-SPEX | Q1 Hotspot | 0.019 | -0.005 | 0.044 | 0.125 |
| TAU-SPEX | Q1 SD Epicenter | 0.020 | -0.007 | 0.047 | 0.153 |
| TAU-SPEX | Q1 % Epicenter | 0.022 | -0.003 | 0.047 | 0.080 |
| TAU-SPEX | Hotspot | 0.022 | -0.007 | 0.050 | 0.133 |
| TAU-SPEX | Global | 0.035 | 0.008 | 0.063 | 0.013 |
| TAU-SPEX | Braak I | 0.040 | -0.021 | 0.102 | 0.200 |
| TAU-SPEX | Current Braak stage | 0.075 | -0.039 | 0.188 | 0.197 |

**Supplementary Table 9: Comparing AUCs for the association between baseline tau-PET and clinical progression across approaches in MCI and mild dementia Aβ+.** For each baseline tau-PET approach, covariate-adjusted AUCs were computed from logistic regression model-predicted probabilities, as described in **Figure 5**. The approach with the highest AUC was identified as the reference and compared against each of the other approaches using DeLong’s test, reporting the AUC difference with 95% confidence intervals and providing two-sided *p*-values for each comparison. AUC = area under the curve; CI = confidence interval; Q1 = quantile 1; ROI = region of interest; SD = standard deviation; TAU-SPEX = tau spatial extent.

| **Best ROI** | **Comparator ROI** | **Δ*R^2^*** | **CI lower** | **CI upper** | ***p*** |
| --- | --- | --- | --- | --- | --- |
| Q1 Hotspot | Global | 0.075 | 0.044 | 0.108 | <0.001 |
| Q1 Hotspot | Braak I | 0.108 | 0.033 | 0.172 | 0.002 |
| Q1 Hotspot | Temporal-meta | 0.005 | -0.021 | 0.031 | 0.429 |
| Q1 Hotspot | % Epicenter | 0.044 | 0.008 | 0.079 | 0.005 |
| Q1 Hotspot | Q1 % Epicenter | 0.012 | -0.001 | 0.025 | 0.031 |
| Q1 Hotspot | Hotspot | 0.047 | 0.003 | 0.091 | 0.020 |
| Q1 Hotspot | SD Epicenter | 0.072 | 0.024 | 0.118 | <0.001 |
| Q1 Hotspot | Q1 SD Epicenter | 0.008 | -0.006 | 0.025 | 0.134 |
| Q1 Hotspot | Current Braak stage | 0.088 | 0.022 | 0.148 | 0.004 |
| Q1 Hotspot | TAU-SPEX | 0.023 | -0.022 | 0.063 | 0.153 |

**Supplementary Table 10: Bootstrapped *R*^2^ comparisons between approaches for the association between longitudinal tau-PET and cognitive decline in CU Aβ+.** Marginal *R*^2^ values were obtained from the linear mixed-effects models relating tau-PET change to mPACC5 change as described in **Figure 6**. The approach with the highest marginal *R*^2^ in the original sample was treated as the reference. Participant-level bootstrap resampling with replacement (1000 iterations; unsuccessful fits excluded) was used to refit the reference model and each comparator model within each bootstrap sample, and the pairwise difference in marginal *R*^2^ (reference − comparator) was computed per iteration. 95% confidence intervals were derived from the bootstrap distribution of *R*^2^ differences. One-sided bootstrap *p*-values were computed as the proportion of iterations in which the *R*^2^ difference was ≤0 (i.e., comparator performed at least as well as the reference). CI = confidence interval; Q1 = quantile 1; ROI = region of interest; SD = standard deviation; TAU-SPEX = tau spatial extent.

| **Best ROI** | **Comparator ROI** | **Δ*R^2^*** | **CI lower** | **CI upper** | ***p*** |
| --- | --- | --- | --- | --- | --- |
| Q1 SD Epicenter | Global | 0.011 | -0.023 | 0.032 | 0.331 |
| Q1 SD Epicenter | Braak I | 0.144 | 0.025 | 0.200 | 0.004 |
| Q1 SD Epicenter | Temporal-meta | 0.007 | -0.036 | 0.045 | 0.381 |
| Q1 SD Epicenter | % Epicenter | 0.126 | 0.028 | 0.157 | 0.001 |
| Q1 SD Epicenter | Q1 % Epicenter | 0.006 | -0.012 | 0.017 | 0.311 |
| Q1 SD Epicenter | Hotspot | 0.143 | 0.033 | 0.175 | 0.002 |
| Q1 SD Epicenter | Q1 Hotspot | 0.002 | -0.024 | 0.015 | 0.574 |
| Q1 SD Epicenter | SD Epicenter | 0.150 | 0.042 | 0.191 | <0.001 |
| Q1 SD Epicenter | Current Braak stage | 0.159 | 0.029 | 0.234 | 0.005 |
| Q1 SD Epicenter | TAU-SPEX | 0.038 | -0.022 | 0.069 | 0.135 |

**Supplementary Table 11: Bootstrapped *R*^2^ comparisons between approaches for the association between longitudinal tau-PET and cognitive decline in MCI and mild dementia Aβ+.** Marginal *R*^2^ values were obtained from the linear mixed-effects models relating tau-PET change to mPACC5 change as described in **Figure 6**. The approach with the highest marginal *R*^2^ in the original sample was treated as the reference. Participant-level bootstrap resampling with replacement (1000 iterations; unsuccessful fits excluded) was used to refit the reference model and each comparator model within each bootstrap sample, and the pairwise difference in marginal *R*^2^ (reference − comparator) was computed per iteration. 95% confidence intervals were derived from the bootstrap distribution of *R*^2^ differences. One-sided bootstrap *p*-values were computed as the proportion of iterations in which the *R*^2^ difference was ≤0 (i.e., comparator performed at least as well as the reference). CI = confidence interval; Q1 = quantile 1; ROI = region of interest; SD = standard deviation; TAU-SPEX = tau spatial extent.

| **Best ROI** | **Comparator ROI** | **ΔAUC** | **CI lower** | **CI upper** | ***p*** |
| --- | --- | --- | --- | --- | --- |
| Current Braak stage | Braak I | 0.001 | -0.018 | 0.020 | 0.937 |
| Current Braak stage | Temporal-meta | 0.005 | -0.028 | 0.037 | 0.777 |
| Current Braak stage | % Epicenter | 0.018 | -0.018 | 0.055 | 0.323 |
| Current Braak stage | Hotspot | 0.021 | -0.016 | 0.058 | 0.266 |
| Current Braak stage | Q1 Hotspot | 0.025 | -0.012 | 0.062 | 0.181 |
| Current Braak stage | Q1 SD Epicenter | 0.027 | -0.010 | 0.063 | 0.149 |
| Current Braak stage | Q1 % Epicenter | 0.031 | -0.007 | 0.070 | 0.111 |
| Current Braak stage | TAU-SPEX | 0.031 | -0.013 | 0.075 | 0.163 |
| Current Braak stage | SD Epicenter | 0.032 | -0.005 | 0.069 | 0.093 |
| Current Braak stage | Global | 0.053 | 0.011 | 0.095 | 0.013 |

**Supplementary Table 12: Comparing AUCs for the association between longitudinal tau-PET and clinical progression across approaches in CU Aβ+.** For each longitudinal tau-PET approach, covariate-adjusted AUCs were computed from logistic regression model-predicted probabilities, as described in **Figure 6**. The approach with the highest AUC was identified as the reference and compared against each of the other approaches using DeLong’s test, reporting the AUC difference with 95% confidence intervals and providing two-sided *p*-values for each comparison. AUC = area under the curve; CI = confidence interval; Q1 = quantile 1; ROI = region of interest; SD = standard deviation; TAU-SPEX = tau spatial extent.

| **Best ROI** | **Comparator ROI** | **ΔAUC** | **CI lower** | **CI upper** | ***p*** |
| --- | --- | --- | --- | --- | --- |
| TAU-SPEX | Global | 0.004 | -0.020 | 0.028 | 0.717 |
| TAU-SPEX | Q1 SD Epicenter | 0.005 | -0.019 | 0.030 | 0.674 |
| TAU-SPEX | Q1 Hotspot | 0.008 | -0.020 | 0.035 | 0.579 |
| TAU-SPEX | Q1 % Epicenter | 0.012 | -0.012 | 0.036 | 0.338 |
| TAU-SPEX | Temporal-meta | 0.028 | -0.010 | 0.066 | 0.145 |
| TAU-SPEX | Current Braak stage | 0.065 | -0.033 | 0.164 | 0.194 |
| TAU-SPEX | % Epicenter | 0.068 | 0.013 | 0.123 | 0.016 |
| TAU-SPEX | Hotspot | 0.085 | 0.023 | 0.148 | 0.008 |
| TAU-SPEX | SD Epicenter | 0.090 | 0.027 | 0.153 | 0.005 |
| TAU-SPEX | Braak I | 0.108 | 0.036 | 0.180 | 0.003 |

**Supplementary Table 13: Comparing AUCs for the association between longitudinal tau-PET and clinical progression across approaches in MCI and mild dementia Aβ+.** For each longitudinal tau-PET approach, covariate-adjusted AUCs were computed from logistic regression model-predicted probabilities, as described in **Figure 6**. The approach with the highest AUC was identified as the reference and compared against each of the other approaches using DeLong’s test, reporting the AUC difference with 95% confidence intervals and providing two-sided *p*-values for each comparison. AUC = area under the curve; CI = confidence interval; Q1 = quantile 1; ROI = region of interest; SD = standard deviation; TAU-SPEX = tau spatial extent.
